# Targeting the chromatin structural changes of antitumor immunity

**DOI:** 10.1016/j.jpha.2023.11.012

**Published:** 2023-11-29

**Authors:** Nian-nian Li, Deng-xing Lun, Ningning Gong, Gang Meng, Xin-ying Du, He Wang, Xiangxiang Bao, Xin-yang Li, Ji-wu Song, Kewei Hu, Lala Li, Si-ying Li, Wenbo Liu, Wanping Zhu, Yunlong Zhang, Jikai Li, Ting Yao, Leming Mou, Xiaoqing Han, Furong Hao, Yongcheng Hu, Lin Liu, Hongguang Zhu, Yuyun Wu, Bin Liu

**Affiliations:** a Weifang People's Hospital, Weifang, Shandong, 261000, China; b School of Life Sciences, Nankai University, Tianjin, 300071, China; c Weifang Traditional Chinese Medicine Hospital, Weifang, Shandong, 261000, China; d Shaanxi Key Laboratory of Sericulture, Ankang University, Ankang, Shaanxi, 725000, China; e Guizhou Education University, Guiyang, 550018, China; f Guizhou Normal University, Guiyang, 550025, China; g School of Medical Imaging, Weifang Medical University, Weifang, Shandong, 261053, China; h Department of Bone and Soft Tissue Oncology, Tianjin Hospital, Tianjin, 300299, China; j Xinqiao Hospital of Army Military Medical University, Chongqing, 400038, China; k Teda Institute of Biological Sciences & Biotechnology, Nankai University, Tianjin, 300457, China

**Keywords:** Antitumor immunity, Chromatin structural, Cancer epigenetics, DNA methylation, Histone modification, Chemotherapy

## Abstract

Epigenomic imbalance drives abnormal transcriptional processes, promoting the onset and progression of cancer. Although defective gene regulation generally affects carcinogenesis and tumor suppression networks, tumor immunogenicity and immune cells involved in antitumor responses may also be affected by epigenomic changes, which may have significant implications for the development and application of epigenetic therapy, cancer immunotherapy, and their combinations. Herein, we focus on the impact of epigenetic regulation on tumor immune cell function and the role of key abnormal epigenetic processes, DNA methylation, histone post-translational modification, and chromatin structure in tumor immunogenicity, and introduce these epigenetic research methods. We emphasize the value of small-molecule inhibitors of epigenetic modulators in enhancing antitumor immune responses and discuss the challenges of developing treatment plans that combine epigenetic therapy and immunotherapy through the complex interaction between cancer epigenetics and cancer immunology.

## Highlights

• This paper mainly studies the effect of Epigenetics regulation on tumor immune cell function.
• The key abnormal Epigenetics processes include DNA methylation, histone modification and their influence in Chromatin structure to modulate tumor immune cells.
• Summarized the research methods of these epigenetic modifications for the antitumor immunity.
• The value of epigenetic modulators and small molecule inhibitors in enhancing anti-tumor immune response was emphasized.
• The challenges of developing a combination of epigenetic and immunotherapy regimens were discussed.

## Graphical abstract

In this review, we mainly review the effects of epigenetic modifications on chromosomal structure, thereby affecting gene expression and their functional realization. Combining cutting-edge epigenetic methods, we summarize in detail how DNA methylation, histone modification, and chromatin 3D structure affect the tumor microenvironment through immune cells to regulate tumorigenesis and tumor metastasis. At the same time, we summarize the effects of genomic topology on immune cells and tumors. This review provides detailed and in-depth methods and insights in epigenetics and tumor immunity, providing doctors and cancer patients with new ideas.

**Figure:**
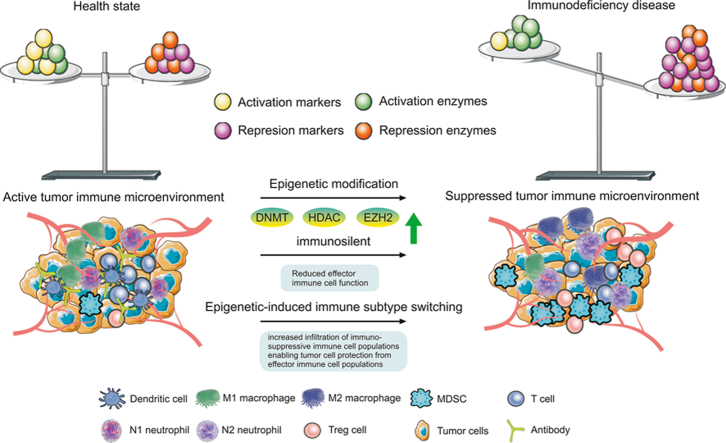

## 1 Introduction

**Fig. 1:**
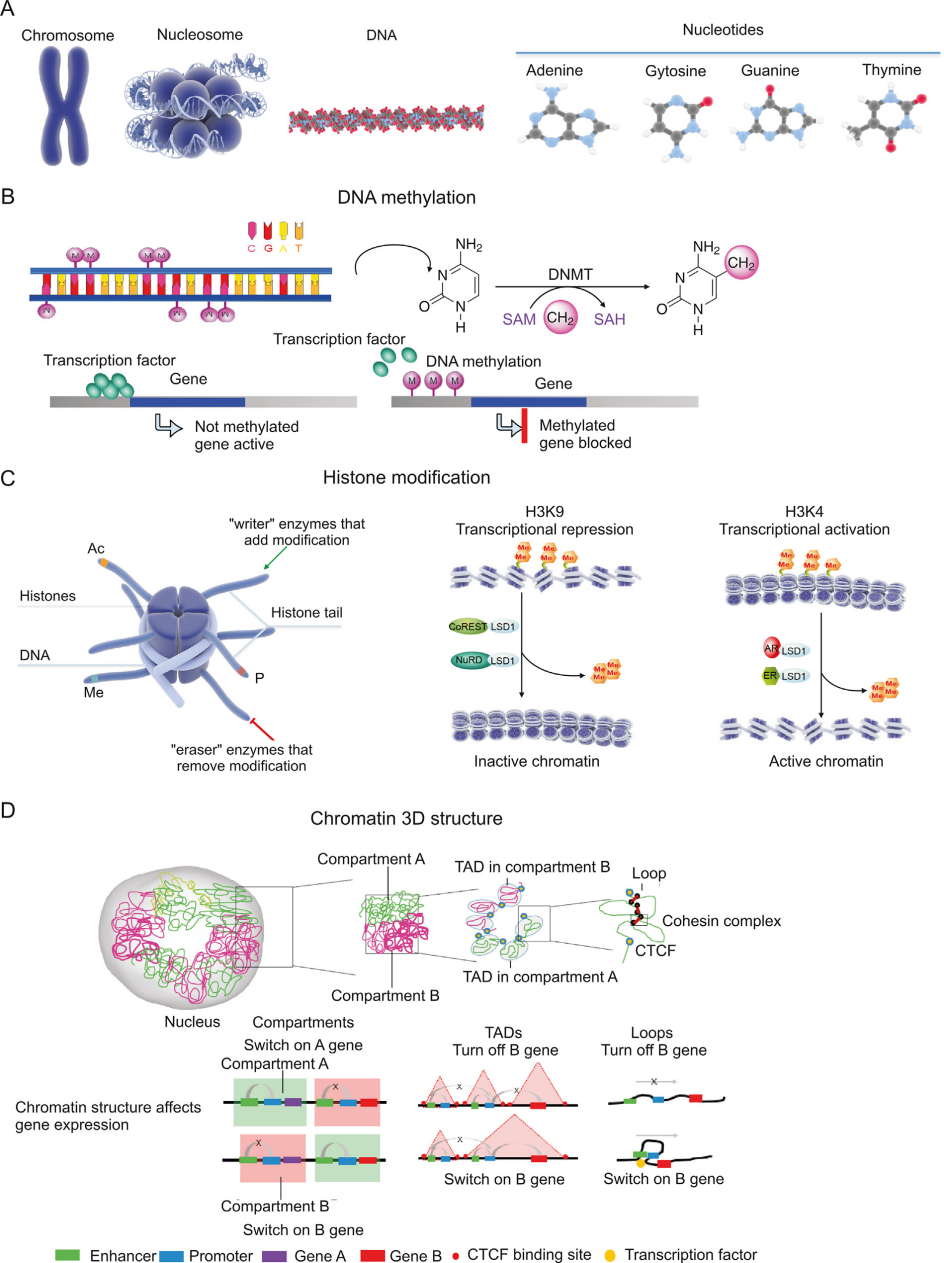
Gene expression changes caused by epigenetic modification. (A) Eukaryotes have multiple chromosomes. Humans have 46 chromosomes totaling 2 m in length. The average length of DNA molecules on each chromosome is about 5 cm, while the diameter of the nucleus is only 5–8 μm; therefore, assembling chromosomes from chromatin DNA requires compressing the chromatin nearly 10,000 times. Chromosomes are composed of multiple nucleosomes, which are connected in series and folded. Nucleosomes, formed from DNA and histones, are the basic structural units of chromatin. Each nucleosome is formed by wrapping 146 bp of DNA around 1.75 loops of the histone octamer. Nucleosome core particles are connected by approximately 50 bp of connecting DNA. DNA comprises deoxyribonucleotides adenine (A) and guanine (G), cytosine (C), and thymine (T). (B) DNA methylation refers to the chemical modification process in which a specific base on the DNA sequence obtains a methyl group by covalent bonding with S-adenosyl methionine (SAM) as a methyl donor under the catalysis of DNA methyltransferase (DNMT). DNA methylation alters the structure of chromatin, inhibits the binding of transcription factors, and prevents transcription. (C) Histone modification refers to the process of histone modifications, such as methylation, acetylation, phosphorylation, adenosylation, ubiquitination, and ADP ribosylation, under the action of related enzymes. Histone modification is reversible by various enzymes, including writers and erasers. Histone modifications occur at different sites of histone amino acids. For example, histone H3 at positions 4, 9, 27, and 36, H4 at position 20 Lys, H3 at positions 2, l7, and 26, and H4 at position 3 Arg are common sites for methylation. Importantly, not all methylation inhibits gene expression; indeed, methylation of lysine residues at position 4 of H3 is associated with gene activation (H3K4me2/3), while methylation of lysine residues at positions 9 and 27 is associated with gene silencing (H3K9me3, H3K27me3). lysine-specific demethylase 1 (LSD1), nucleosome remodelling and deacetylase (NuRD), repressor element 1 silencing transcription factor corepressor (CoREST), nuclear receptor (NR), and estrogen receptor (ER) are involved in histone modification. (D) The development of chromatin interaction detection technology has promoted the study of the 3D structure of chromatin. In a crowded nuclear space, chromatin folds into specific hierarchical structures, including the A/B compartment, topologically associated domain (TAD), and chromatin loop (loop). During the process of cell fate transition, drastic changes in the chromatin folding state are closely related to changes in gene expression. For example, genes located in the A department can undergo transcriptional activation, while inhibition of transcription occurs in genes in the B department. Only DNA elements within the same TAD can interact. Enhancer promoters form loops to initiate gene expression. SAH: S-adenosyl-l-homocysteine; Ac: acetylation; Me: methylation; P: phosphorylation; CTCF: CCCTC-binding factor.

Deoxyribonucleic acid (DNA), which includes the four nitrogenous bases adenine (A), guanine (G), cytosine (C), and thymine (T), is the main genetic material in eukaryotic cells (Fig. 1A) [1]. In DNA, A–T and G–C base pairs and nitrogen–hydrogen and oxygen–hydrogen bonds are reused to form a DNA double helix structure [1]. Most eukaryotic chromosomes contain packaging proteins that combine with DNA molecules to form nucleosomes with the help of molecular chaperones. A series of nucleosomes are regularly wrapped and stacked together to form chromosomes [2]. Epigenetic research includes DNA modification (for example, DNA methylation), histone modification, noncoding ribonucleic acid (RNA) regulation, chromatin remodeling, and nucleosome localization. Genetics and epigenetics involve the study of how changes in these genetic materials cause growth, development, and disease. Studies on antitumor immunity have focused on the immune system and how it prevents tumor cell transfer and tumor formation [3]. In this review, we conduct an in-depth analysis of the relationship between epigenetics and tumor immune suppression, with a focus on the role of DNA methylation (Fig. 1B) [4], histone acetylation, and histone methylation in regulating the antitumor immune response (Fig. 1C) [5,6]. The covalent modification of histones and nucleic acids jointly regulates the chromatin structure, for the core of epigenetics. The changes in the chromatin structure will affect gene expression, thus changing the state of cells. Epigenetic modification is reversible and dynamically regulated; that is, the chromatin structure can be dynamically changed. For example, open euchromatin can be converted into tight heterochromatin (Fig. 1D) [7]. In open chromatin, gene expression is usually active, whereas in heterochromatin, it is usually silent. Genetic and epigenetic changes are important factors in carcinogenesis, tumor progression, and metastasis that alter the expression of tumor-related genes [7].

Epigenetic changes promote carcinogenesis by affecting the pathways of multiple oncogenes and tumor suppressor genes in a wide range of cells [8], and by affecting the activation, differentiation, and functional fate of immune cells (e.g., T cells and natural killer cells, also called NK cells), as a cancer monitoring mechanism [9]. Some epigenetic changes occur early on, before tumor development begins [10]. Indeed, a recent study showed that epigenetic programming induced by the tissue environment initiated tumorigenesis [11]. Moreover, Feinberg et al. [12] proposed the epigenetic progenitor cell origin of human cancer. These findings provide a strong theoretical basis for the application of epigenetic drugs in cancer treatment. Epigenetic therapy regulates the expression of related genes in immune cells, other normal cells, or cancer cells, with the aim of affecting the fate of these cell populations [13]. Epigenetic drugs are chemicals that act on the epigenome of cells to exert their functions, and include DNA methyltransferase (DNMT) inhibitors, DNA demethylase, histone deacetylase (HDAC), histone acetyltransferase (HAT), histone methyltransferase (HMT), histone demethylases (HDMs), and other related enzymes. These unique enzymes can write or erase epigenetics, where they are termed “writers” or “erasers,” respectively [14]. In-depth research on epigenetics has led to the development of various small molecule drugs that alter epigenetic modifications and have been applied in the clinical treatment of tumors. Small-molecule inhibitors of DNMT, commonly known as hypomethylation agents, can be considered to be “scavengers” of methylation modification [15]. The inhibition of methylation is the most widely used epigenetic therapy for cancer, mainly for the treatment of myelodysplastic syndrome (MDS) and acute myeloid leukemia (AML) [3,16]. Moreover, 5-Aza, 5-Aza-2′-deoxycytidine, and SGI-110 are cytidine analogs, which irreversibly separate DNMT from DNA, resulting in global DNA hypomethylation [4,16]. The direct antitumor effects induced by DNMT inhibitors, such as apoptosis, cell cycle arrest, and differentiation, have been attributed to changes in gene expression related to DNA methylation. Indeed, after treatment with DNMT inhibitors and hypomethylation agents, the enrichment of immune-related pathways can increase the expression of genes related to antigen presentation, as well as immunostimulatory molecules such as CD80, CD86, and CD40.

In this review, we summarize the process of chromatin structure changes caused by the epigenetic modification of immune cells during cancer progression and treatments. Additionally, we discuss potential innovative strategies for targeting immune cells and cancer cells with epigenetic modulators to develop more effective targets in combination immunotherapy.

## 2 Gene expression changes caused by epigenetic modification

DNA methylation is one of the earliest discovered and most deeply studied epigenetic regulatory mechanisms. In a broad sense, DNA methylation refers to the chemical modification process in which a specific base on the DNA sequence obtains a methyl group through covalent bonding with S-adenosyl methionine (SAM) as a methyl donor under the catalysis of DNMT (Fig. 1B) [15]. DNA methylation can occur at the C-5 position of cytosine, the N-6 position of adenine, and the N-7 position of guanine. DNA methylation mainly refers to the methylation process of the fifth carbon atom on cytosine in the CpG dinucleotide [17]. The product of this process is called 5-methylcytosine (5-mC), which is the main form of DNA methylation in eukaryotic organisms (e.g., plants and animals) and the only form of DNA methylation found in mammals. DNA methylation, as a relatively stable modification state, can be passed on to newborn offspring DNA with the DNA replication process under the action of DNA methyltransferase, which serves as an important epigenetic mechanism [4]. DNA methylation inhibits gene expression in two ways. In the first way, DNA methylation can promote DNA to condense into tightly arranged heterochromatin, prevent the combination of transcription factors (such as E2F2) and transcription initiation sites, and interfere with gene transcription [18]. In the second way, DNA methylation recruits other transcription co-inhibitors (e.g., HDACs, HMTs, DNMTs) after the methylated CpG is combined with the CpG methylation binding protein family (MeCPs) [19]. These proteins then package DNA to form a dense chromatin structure, inhibit the binding of transcription factors, and prevent transcription (Fig. 1B) [15].

In mammalian genomes, histones can have many modified forms. A nucleosome consists of a histone octamer composed of two H2A, two H2B, two H3, and two H4, with 147 bp of DNA wrapped around it [20]. The core part of histones that make up nucleosomes is roughly uniform, although the free N-terminal of histones can be subject to various modifications, including acetylation, methylation, phosphorylation, ubiquitination, and ADP ribosylation, all of which affect the transcription activity of genes. In this review, we consider the influence of the chromatin structure and introduce the methylation and acetylation of chromatin in detail (Fig. 1C) [21].

Histone methylation is accomplished by HMT. Methylation can occur on the lysine and arginine residues of histones; the lysine residues can undergo single, double, and trimethylation, while the arginine residues can undergo single and double methylation. These different degrees of methylation greatly increase the complexity of histone modification and regulation of gene expression [22]. The action site of methylation is on the side chain N atoms of lysine (Lys) and arginine (Arg). The 4th, 9th, 27th, and 36th positions of histone H3, the 20th Lys of H4, the 2nd, 7th, and 26th positions of H3, and the 3rd Arg of H4 are all common sites of methylation [23]. Research has shown that histone arginine methylation is a relatively dynamic marker, and arginine methylation is related to gene activation, while the loss of H3 and H4 arginine methylation is related to gene silencing [24]. In contrast, lysine methylation seems to be a relatively stable marker in gene expression regulation. For example, the methylation of lysine residues at the 4th position of H3 is related to gene activation, while the methylation of lysine residues at the 9th and 27th positions is related to gene silencing [25]. Additionally, the methylation of H4-K20 is related to gene silencing, and the methylation of H3-K36 and H3-K79 is related to gene activation [26]. However, it should be noted that the number of methylations is related to the degree of gene silencing and activation.

Histone acetylation mainly occurs at the relatively conservative lysine position at the N-terminal of H3 and H4, and is coordinated by histone acetyltransferase and histone deacetylase. Although the nucleosome has multiple sites that can serve as acetylation sites, histone acetylation and deacetylation of specific gene sites are conducted in a non-random and location-specific manner [27]. Acetylation may regulate gene transcription by affecting histone charge and interacting with proteins. In the early classification of chromatin and its characteristic components, some researchers highlighted that the heterochromatin domain histones showed low acetylation, while those of the euchromatin domain showed high acetylation [28]. Recent studies have found that some HAT complexes contain common transcription factors and some HDAC complexes contain proven repressor proteins. These findings support the view that high acetylation is related to the activation of gene expression, and low acetylation is related to the inhibition of gene expression.

Relatively speaking, histone methylation modification is the most stable type of histone modification, making it optimal for stable epigenetic information. Acetylation modification is highly dynamic and prone to change, making it similar to many other unstable modification methods, including phosphorylation [29], adenosylation [30], ubiquitination [31], and ADP ribosylation [32]. These modifications can flexibly affect the structure and function of chromatin, while combinations of various modifications allow chromatin to exert its regulatory function. Owing to their functional importance, these modifications are often specifically recognized as histone codes. Histone codon combinations vary greatly, so histone covalent modification may be a more refined mode of gene expression. Additionally, previous studies have found that the ubiquitination of H2B can affect the methylation of H3K4 and H3K79, further highlighting the correlation between various modifications [33].

The process of cell fate transformation is accompanied by a dynamic change in the three-dimensional (3D) structure of chromatin [34]. In recent years, the development of chromatin interaction detection technology has promoted the study of the 3D structure of chromatin. In the crowded nuclear space, chromatin folds to form specific hierarchical structures, including the A/B department, topologically associated domain, and chromatin loops. In the process of cell fate transformation, the drastic change in the chromatin folding state is closely related to the change in gene expression. It is important to determine the formation mechanism and change rule of the 3D chromatin structure to better understand gene expression regulation and cell fate transformation (Fig. 1D) [35].

Researchers have found that each chromosome in the nucleus tends to occupy independent and non-overlapping regions, known as chromosomal boundaries, with the chromatin within the boundaries forming different compartment structures [36]. According to their different transcriptional activities, these compartments can be divided into an active transcription A compartment and a transcription-inhibited B compartment. The interaction of chromatin in the same compartment is high, while that between different compartments is low [37]. With the improvement of sequencing accuracy, researchers have found that approximately 1 Mb of DNA in the interior of the region forms a smaller spatial structure called the topologically associated domain (TAD) [38]. The TAD generally contains 8–10 genes, and its internal DNA elements form a relatively close interaction, while the chromatin interaction between TADs is lower. The border of adjacent TADs is bound with chromatin structural proteins, such as the protein complex of CCCTC binding factor (CTCF) and mucin, which serve to organize the chromatin structure and isolate the interaction between two adjacent TADs. The enhancer–promoter that initiates gene transcription is usually located in the same TAD. The TAD also contains chromatin rings, such as enhancer–promoter loops, which are necessary to initiate downstream gene expression (Fig. 1D) [39].

### 2.1 Epigenetic modification affects the tumor microenvironment by determining the fate of immune cell differentiation

**Fig. 2:**
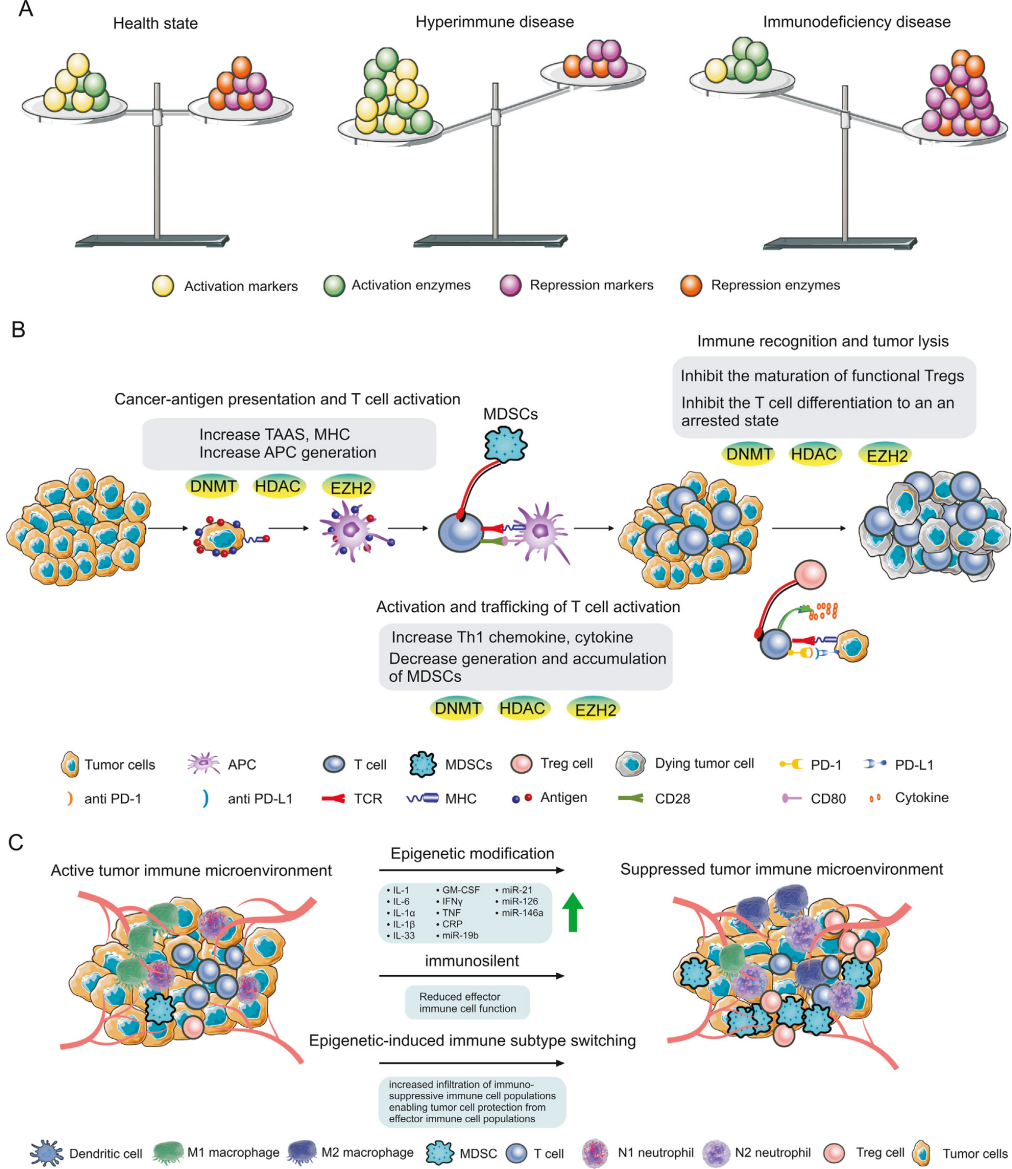
Epigenetic changes in the tumor immune microenvironment. (A) The balance of the immune microenvironment is particularly important for human health, with low immunity leading to colds, acquired immunodeficiency syndrome (AIDS), tumors, and other diseases, and excessive immunity known to be associated with autoimmune diseases such as systemic lupus erythematosus, rheumatoid arthritis, and scleroderma. (B) The immune tumor microenvironment contains all cells involved in the immune response of the body. These cells include cytotoxic T lymphocytes (CTL), natural killer (NK) cells, myelosuppression cells (MDSCs), regulatory T cells (Tregs), and tumor-associated macrophages (TAMs) that play an immunosuppressive role. Epigenetic modification plays an important regulatory role in the development of the tumor immune microenvironment [43]. (C) Immunosuppressive epigenetic modification of the tumor microenvironment leads to a sharp decline in immune function, resulting in a significant reduction in the cytotoxic activity of effector immune cell subsets, including T cells, NK cells, macrophages, and dendritic cells. In the activated immune microenvironment, these effector cells are responsible for purging malignant tumor cells. Immunosuppressive MDSCsand Tregs are significantly increased by adverse epigenetic modification, while effector cells such as neutrophils and macrophages appear to shift to immunosuppressive N2 and M2 states, respectively, to promote tumorigenesis. DNMT: DNA methyltransferase; HDAC: histone deacetylase; EZH2: enhancer of zeste homolog 2; TAAs: tumor-associated antigens; APC: antigen-presenting cell; TCR: T cell receptor; MHC: major histocompatibility complex.

Immunity largely consists of cellular and humoral immunity, comprising T cells and immunoglobulins, respectively. Once foreign substances (e.g., bacteria, viruses, and various foreign proteins) invade the body and endanger health, the immune cells surround, decompose, swallow, and finally eliminate them, while immunoglobulins are produced in large quantities to strengthen the response against the invaders [40]. Human health needs immunity to maintain a relatively balanced state, and insufficiency of the immune system may cause colds, human immunodeficiency virus (HIV) invasion, tumor generation, and other disease occurrences. However, excessive immunity, when immune cells are overactivated, also causes harm to the human body. Excessive immunity can lead to autoimmune diseases such as systemic lupus erythematosus, rheumatoid arthritis, and scleroderma (Fig. 2A).

The immune tumor microenvironment is known as the “seventh feature” of tumors, and is composed of innate immune cells, adaptive immune cells, cytokines, and cell surface molecules. These immune components constitute a complex regulatory network, which plays an important role in the occurrence and development of tumors [19]. Epigenetic modification affects the differentiation and fate of immune cells, thus affecting the occurrence and development of tumors. The immune tumor microenvironment contains all immune cells that participate in the immune response [41], including cytotoxic T lymphocytes (CTL) and NK cells, as well as the immunosuppressive myeloid-derived suppressor cells (MDSC), regulatory T cells (Treg) [42], and tumor-associated macrophages (TAMs) (Fig. 2B) [43]. The role of the immune system in tumor monitoring and cancer treatment has been fully established. The immune system can recognize and eliminate tumor cells through the innate and adaptive arms of the immune response. After tumorigenesis, tumor-associated antigens (TAAs) are often expressed in large quantities and presented to antigen-presenting cells (APCs) through the histocompatibility complex (MHC) [44]. T cells recognize endogenous antigen peptides presented by MHC molecules. The activated and differentiated effector cells are Tc (CTL) cells, which can specifically kill target cells and are the main effector cells of cellular immunity. Simultaneously, CD28, the receptor of the costimulatory molecule B7, is expressed by T cells. B7 molecules include B7-1 (CD80) and B7-2 (CD86), which are mainly expressed by professional APCs. The costimulatory signal generated by the combination of CD28 and B7 plays an important role in the activation of T cells, inducing T cells to express anti-apoptotic proteins, stimulating T cells to synthesize IL-2 and other cytokines, and promoting the proliferation and differentiation of T cells [45]. In cancer, myeloid cells can differentiate into subtypes with antitumor functions (e.g., “M1” macrophages) or tumor-promoting functions (e.g., MDSCs). M1 macrophages produce chemokines that are essential for the recruitment of lymphocytes (e.g., chemokine (C-X-C motif) ligand 9 (CXCL9) and CXCL10) and cytokines (e.g., tumor necrosis factor (TNF)) with antitumor functions [46]. On the contrary, tumor-promoting “M2” macrophages and MDSCs inhibit antitumor immune responses through a variety of mechanisms, including inhibiting T cell activation. This differentiation is highly plastic and can be modified with epigenetic modifiers to promote antitumor immunity [47]. Activated T cells express programmed death 1 (PD-1) on their surface, while tumor cells express immunoglobulin-like molecules, such as programmed cell death-ligand 1 (PD-L1). After binding of PD-1/PD-L1, a molecular signal is generated, which reduces the activity of immune cells, thereby blocking the tumor immune response. The tumor uses this as an immune evasion strategy to survive; however, the use of antibodies to PD-1 and PD-L1 can block this connection and improve cellular immunity [43]. Moreover, regulatory T cells (Tregs), which can inhibit the activation of effector T cells through cell–cell contact and *in vivo* regulation, is recognized as another accomplice of tumor immune escape. Activated T cells release the cytokines perforin, serine ester alcohol, type I interferons (IFN), and TNF, which promote the death and lysis of tumor cells [48].

### 2.2 Epigenetic changes in tumor immune microenvironment

TAA) is essential for the occurrence of tumor immunity. One of the most effective strategies for tumor cell escape is to destroy antigen presentation, which is the initial step of the immune cycle and plays an important role in tumor immunotherapy. Co-stimulatory genes, tumor antigens, and major MHC are essential for the activation of effector T cells [49,50]. Existing studies have shown that CD8 cells with an antigen-specific T cell level close to 1% may be necessary to establish an effective antitumor response. However, current research shows that the antigen preset level of most cancer cells is low or even not, that is lower than the perception limit of the immune system, thus causing tumor cell immune escape [51]. The epigenetic regulation can regulate the expression of TAAs in different tumor cells. In clinical applications, DNA methyltransferase inhibitor (DNMTi) treatment can improve tumor immunogenicity and immune recognition by promoting the re-expression of TAAs [52]. Drugs that act on epigenetic modification enzymes have been widely used in the clinical treatment for patients with tumor (Table 1) [53,54]. DNA methylation is considered to be the main mechanism of regulating the expression of cancer testicular antigen (CTA) [55]. CTA is a family of tumor-related antigens expressed on tumors. DNMTis up-regulate MHC class I and II and have been found in many cancers [56]. Decitabine (5-Aza-′-deoxycytidine, DAC) is an effective inhibitor of DNA methylation [57]. The treatment of chronic lymphocytic leukemia (CLL) cell lines with DAC *in vitro* can upregulate the expression of MHC class I and MHC class II [58]. DAC treatment also up-regulated the surface expression of MHC class I and showed an increase in the release of IFN-g from tumor-specific CTLs [59]. Treatment of RM-1 prostate cancer cells with another epigenetic-modified 5-azacytidine (5-AzaC) can enhance the expression of CTA [60]. Additionally, 5-AzaC enhances the pro-inflammatory function of dendritic cells (DCs) by increasing MHC class I, MHC class II, CD80, CD86, CD205, and CD4073 [3]. Many studies have shown that exposure to demethylating agents can lead to the expression of multiple CTAs in a variety of tumor cell lines, including melanoma and malignant glioma. New research has shown that enhancer of zeste homolog 2 (EZH2) is activated or overexpressed by mutations in melanoma and other tumors, resulting in the silencing of antigen presentation-related genes and cancer suppressor genes [61]. Regarding the potential function of epigenetic regulators in many aspects of the tumor microenvironment and immune cycle, epigenetic drugs such as 5-AzaC may induce immunogenic cell death (ICD) of cancer cells, enhance the expression of various TAAs, MHC molecules, and the generation of APCs, thus enhancing the recognition of tumor target cells by immune cell activation and effector T cells. DNMTi, histone deacetylase inhibitors (HDACi), and histone methyltransferase inhibitors (HMTi) (EZH2 and G9a) have demonstrated these biological effects [62].

**Table 1:** Agents that act on epigenetic modification enzymes are used in patients with cancer.

Name	Mechanisms of action	Clinical applications
Azacitabine	DNMT inhibitor	AML; CMML; MDS
Decitabine	DNMT inhibitor	AML; CMML; MDS
Vorinostat	HDAC inhibitor	CTCL
Romidepsin	HDAC inhibitor	CTCL and PTCL
Belinostat	HDAC inhibitor	PTCL
Panobinostat	HDAC inhibitor	Multiple myeloma
Chidamide	HDAC3 inhibitor	PTCL
Enasidenib	IDH2 inhibitor	AML
Ivosidenib	IDH1 inhibitor	AML

DNMT: DNA methyltransferase; HDAC: histone deacetylase; IDH: isocitrate dehydrogenase; AML: acute myeloid leukemia; CMML: chronic myelomonocytic leukemia; MDS: myelodysplastic syndromes; CTCL: cutaneous T cell lymphoma; PTCL: peripheral T-cell lymphoma.

The goal of cellular immunity is to transfer effector T cells to tumor sites and release cytokines. A previous study showed that the demethylase trimethylation of lysine 27 on the component of polycomb inhibitor complex 2 (PRC2) and histone H3 (H3K27me3) inhibited the transport of effector T cells by downregulating the expression of CXCL9 and CXCL1 [63]. Moreover, PRC2-mediated epigenetic silencing has been found to be associated with the inhibition of effector T-cell transport and the improvement of anti-PD-L1 treatment in mice. EZH2-mediated DNA methylation associated with DNMT1 and H3K27me3 may inhibit the expression of the type 1 T helper cell (TH1) chemokines CXCL9 and CXCL10, as well as the subsequent transport of effector T cells to the tumor microenvironment [64]. The application of epigenetic drugs can increase the penetration of effector T cells into tumors and improve the clinical efficiency of PD-L1 checkpoint blockade. Therefore, epigenetic silencing of TH1 chemokine is an important mechanism of immune escape.

The tumor microenvironment is usually associated with immunosuppressive cells, which have negative effects on the activation, migration, and proliferation of T cells. MDSCs, immature DCs, macrophages, and Tregs [65] are known to have key roles in the tumor microenvironment [66]. Many Treg-specific epigenetic characteristic genes, such as *CTLA4*, *IKZF4* (EOS), and *TNFRSF18* (GITR), show complete demethylation, which allows fork box P3 (FOXP3) T cells to obtain Treg characteristic gene expression, lineage stability, and specific immunosuppressive activity [67]. EZH2 plays a crucial role in the differentiation of peripheral Tregs, which may also contribute to the efficiency of PD-1 immunotherapy [68]. This finding suggests that the effect of epigenetic modification on macrophages may also trigger effects on T-cell infiltration. In another study, immunosuppressive MDSCs were reduced by the DNA demethylating agent 5-AzaC in the tumor microenvironment to promote an antitumor immune response. Epigenetic reprogramming is closely related to patient survival via its effects on T cell transport and infiltration. Epigenetic drugs may target multiple types of immune cells, resulting in reduced production and accumulation of MDSCs [69] and inhibiting the differentiation and function of Tregs (e.g., enhancer of zeste homolog 2 inhibitors (EZH2i)) [70]. Therefore, epigenetic drugs can be considered an effective method to promote the tumor infiltration of T lymphocytes to improve the efficiency of current immunotherapy.

Immune cell transformation, inflammation, and immunosuppression are the driving factors inducing tumor progression. Many characteristics of cancer require immune regulation, which is an important step to achieve effective progress. Unfavorable surface modification will lead to a sharp decline in immune function, resulting in a significant reduction in cytotoxic activity of effector immune cell subsets, including T cells, NK cells, macrophages, and dendritic cells (DCs) [71]. In the activated immune microenvironment, these effector cells are responsible for clearing malignant tumor cells. Epigenetic modification plays a key role in tumor formation by altering the function of effector immune cells and inducing the transformation of tissue specificity to more immunosuppressive cell populations. Many studies have shown that immunosuppressive MDSCs [72] and Tregs [73] are significantly increased under adverse epigenetic modifications, and, in some cases, contribute to tumor progression. These immune components are also essential for establishing pre-metastatic niches in many cancer types [74]. Additionally, under the effect of epigenetic modification, effector cells, such as neutrophils and macrophages, seem to turn to immunosuppressive N2 and M2 states [75], respectively, which have been shown to promote tumorigenesis of various cancer types, while more direct studies have shown that they are necessary for age-related tumorigenesis. Immunosuppressive N2 and M2 states have been proven to destroy the acute inflammatory response to malignant tissues, induce the infiltration of immunosuppressive MDSCs and Tregs, and promote the secretion of anti-inflammatory components such as cytokines and chemokines (Fig. 2C) [76].

### 2.3 Effect of DNA methylation on tumorigenesis and development

The methylation pattern of DNA in the genome is realized by DNA methyltransferases. DNA methylation enzymes are divided into two categories: maintenance DNA methyltransferases (Dnmt 1) and de novo methylases (DNMT3a and DNMT3b) [77]. Ten-eleven translation protein is a kind of protein existing in organisms α-Ketoglutaric acid (α-KG) and Fe^2+^dependent dioxygenase having a catalytic domain at the end near C, which has three metal ions (Fe^2+^). At the binding site of KG, there is a cysteine-rich region in front of the catalytic domain [78]. Ten-eleven-translocation (TET) proteins can catalyze the conversion of 5-methylcytosine (5mC) to 5-hydroxymethylcytosine (5hmC), which is an important enzyme in the process of DNA demethylation. Mutation of the *TET* gene can cause many tumors, especially hematopoietic system tumors [79].

2-Hydroxyglutaric acid (2-HG) is a type of tumor binary stone produced by changes in the morphology and function of mutated isocitrate dehydrogenase (IDH) enzymes IDH1 and IDH2. These enzymes are frequently mutated in AML, vascular immunoblastic T-cell lymphoma, and glioma. 2-HG functional inhibition α-Ketoglutarate dependent dioxygenases include the TET family and Jumanji family of histone demethylase [80]. Patients with AML with IDH1/IDH2 mutations are characterized by global DNA hypermethylation and genomic instability, which promotes malignant transformation. Previous studies have shown no significant correlation between IDH1/IDH2 mutation and immune cell infiltration and PDL1 expression in glioma. IDH1/IDH2 mutation reduces STAT1 expression, which may be caused by excessive methylation of the STAT1 promoter and a reduction in the induction of MHC class I and CXCL9/CXCL10 [81]. Aberrant DNA methylation may be a major carcinogenic event, which may change the antitumor immune response and further aggravate tumorigenesis [82].

**Fig. 3:**
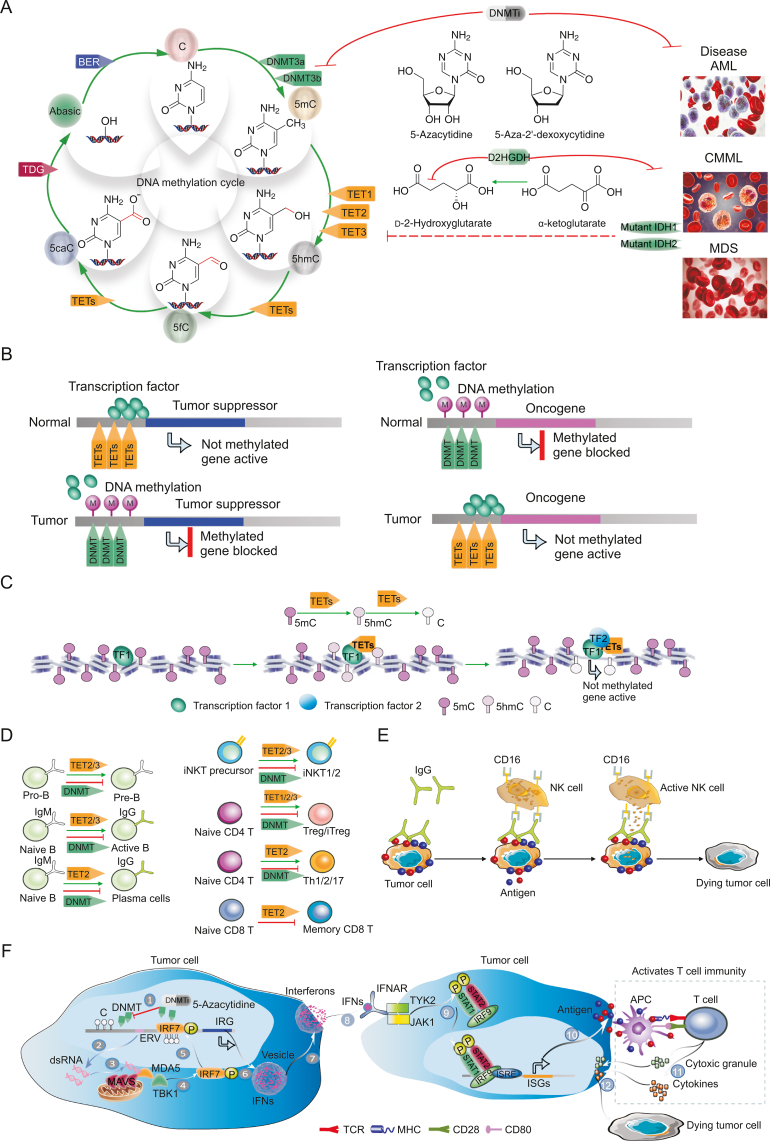
Effect of DNA methylation on tumorigenesis and development. (A) In DNA, the 5-carbon of cytosine can be methylated to form 5-methylcytosine (5mC) through the DNA methyltransferases (DNMT3a and DNMT3b). A small molecular analog of cytosines, such as 5-azacytidine and 5-aza-2′-deoxycytidine, binds to DNA and inhibits DNMT attachment to DNA, resulting in hypomethylation. These hypomethylation reagents are used to treat human cancers, including acute myeloid leukemia and myelodysplastic syndromes. Subsequently, 5mC is gradually oxidized to 5-hydroxymethylcytosine (5hmC), which undergoes the ten-eleven-translocation (TET) of methylcytosine dioxygenases TET1, TET2, and TET3. However, the TET enzyme-dependent metabolic cofactor α-Ketoglutaric acid can be inhibited by the function of d-2-hydroxyglutaric acid, which is produced by mutant citrate dehydrogenase (IDH). As shown in the pink box, genes that regulate the DNA methylation cycle are often mutated in human cancers. (B) DNMT and TET regulate gene expression in tumor cells. (C) Binding of transcription factors can cause significant modifications in methylation. Transcription factors recruit TETs to convert the methylated 5mC to 5hmC, which ultimately converts to C, before binding of additional transcription factors to this site, ultimately initiating gene expression. (D) Effects of TET protein regulation on lymphatic development and function in mice. Using Mb1-cre Tet2/3-deficient mice, it has been shown that Tet2 and Tet3 partially regulate the transformation from pre B cells to pro B cells by enhancing the rearrangement of immunoglobulin light chains. Moreover, the removal of Tet2 with Vav-Cre and Cd19-Cre result in the proliferation of B cells in the germinal center, while the deletion of Tet2, driven by Vav-Cre, results in reduced plasma cell differentiation. Cd4-cre-Tet2/3-deficient mice exhibit a tendency to differentiate into iNKT17 cells, partly due to reduced expression of Tbx21 and Zbtb7b, as well as a significant T-cell dependent expansion of affected T cells. Tet protein promotes the differentiation of immature CD4 T cells into iTregs *in vitro* through demethylation of the Fox3 enhancer CNS2. CD4 T cells from Cd2 Cre Tet2-deficient mice show impaired Th1, Th2, and Th17 differentiation and cytokine production. Moreover, in response to lymphocytic choroidal meningitis virus infection, CD8 memory cell differentiation has been shown to increase in Cd4 Cre Tet2 deficient mice. (E) During antibody-dependent cell-mediated cytotoxicity (ADCC), the Fab fragment of the antibody binds to the antigen epitope of virus infected cells or tumor cells, and its Fc fragment binds to the FcR on the surface of killer cells (e.g., natural killer (NK) cells, and macrophages) to mediate the direct killing of target cells. Methylation modification affects the differentiation of NK cells and determines the production of IgG antibodies by immune cells, thus playing a decisive role in tumor immunity. (F) DNA methylation is also widely used in eukaryotes to inhibit the transcription of transposable elements that have been integrated into the host genome, such as endogenous retroviruses (ERVs). After promoting the re-expression of ERV in tumor cells, hypomethylation drugs stimulate a natural antiviral response (so-called viral mimicry). BER: base excision repair pathway; TDG: thymidine DNA glycosylase; 5caC: 5-carboxylcytosine; 5fC: 5-formylcytosine; D2HGDH: D-2-hydroxyglutarate; AML: acute myeloid leukemia; CMML: chronic myelomonocytic leukemia; MDS: myelodysplastic syndrome; C: cytosine C; TF: transcription factor; MAVS: mitochondrial antiviral-signaling protein; IFNs: interferons; APC: antigen presenting cell.

The human TET protein family comprises three members: TET1, TET2, and TET3. TET1 was named following its identification in a patient with leukemia, while TET2 and TET3 were successfully cloned from a fetal brain cDNA library. TET1 was first identified as an enzyme that catalyzes the hydroxylation of 5-mC. Further research found that the three TET proteins can convert 5mC to 5hmC. The discovery of the biological function of the TET protein family provides a new understanding of the demethylation mechanism of 5-mC, indicating that 5hmC is an important intermediate in the demethylation process of 5mC [83]. 5hmC can further generate 5-hydroxymethyluracil (5hmU) under the catalysis of deaminase AID, while 5hmU can be recognized and removed by thymidine DNA glycosylase (TDG), and finally, the site is converted into cytosine through base excision repair pathway (BER) to realize DNA demethylation. In contrast, 5hmC can also realize the demethylation effect of 5-mC through other mechanisms. It has also been found that 5-formylcytosine (5fC) and 5-carboxycytidine (5caC) contribute to the process of 5-mC demethylation. Following conversion of 5mC to 5hmC by TET protein, it continues to catalyze the conversion of 5hmC to 5fC and 5caC, before the formation of cytosine at this site is realized under the BER mechanism. The addition of cytosine-modified DNMT can achieve dynamic balance of three base types, including C, 5mC, and 5hmC, at the same site on DNA, which can be regarded as a mechanism of DNA reversible methylation (Fig. 3A) [84].

DNA methylation is a normal and universal modification in eukaryotic cells and is also the main epigenetic form of mammalian gene expression regulation [85]. Although the nucleotide sequence and composition of DNA methylation did not change, the gene expression was affected. During methylation modification, the base of the modified site can be the N-6 position of adenine, the N-4 position of cytosine, the N-7 position of guanine, or the C-5 position of cytosine [86]. In all cases, the modification is catalyzed by different DNA methylation enzymes, but most of them occur on the CpG island of the gene promoter region. During DNA methylation, cytosine protrudes from the double helix of DNA and into a gap where it can bind to enzymes. Under the catalysis of cytosine methyltransferase, the active methyl is transferred from S-adenosylmethionine to cytosine 5 to form 5mC [87]. Methylation of the gene promoter region can lead to transcriptional silence. In the process of tumor occurrence and development, methylation of the tumor suppressor gene promoter region and demethylation of the proto-oncogene promoter region may occur, resulting in inhibition of tumor suppressor gene transcription and false activation of proto-oncogene, and ultimately promoting tumor formation and development (Fig. 3B) [88]. More recently, researchers have speculated that a combination of transcription factors causes obvious modification of methylation, as well as the loose arrangement of methylation sites. Upon combining of transcription factors, they recruit TETs to convert the methylated 5mC into 5hmC, and 5hmC finally into C, before more transcription factors are combined to this site, and finally initiate gene expression (Fig. 3C) [89].

From bone marrow progenitor cells to peripheral memory cells and plasma cells, the B cell genome undergoes progressive demethylation after differentiation [90]. TET2 is one of the most commonly mutated genes in B-cell lymphoma (6%–12%), a malignant tumor originating from B cells in the germinal center, suggesting that TET plays an important role in B cell differentiation [91]. Indeed, when Mb1-Cre is used to knock out Tet2 and Tet3 in early B cells during bone marrow development, differentiation of B cells stops when transitioning from the pre B stage to the pro B stage (Fig. 3D) [92]. One function of TET in the early development of B cells is that it regulates Ig κ during the arrangement of light chain genes, which pair with the rearranged Ig heavy chain to form a complete B cell receptor. TET is passed through Ig κ and promotes its DNA demethylation and chromatin accessibility to regulate Ig κ rearrangement.

In thymocytes and peripheral T cells, the expression levels of Tet2 and Tet3 are higher than those of Tet1, and they are responsible for most 5hmC modification in these cells. Knocking out Tet2 alone in hematopoietic cells or T cells (Cd4 Cre) using Cd2 Cre does not result in any significant defects in T cell development [93], indicating that Tet3 can compensate for the loss of Tet2. Indeed, the absence of Tet2 and Tet3 in T cells of mice using Cd4 Cre resulted in a large-scale lymphoproliferative phenotype of splenomegaly and lymph nodes, and the mice died at 8 weeks of age [94]. At 3–4 weeks of age, the number of thymocytes in young Tet2/3 knockout mice was decreased, the percentage of Cd4+CD8+double positive cells was decreased, and the percentage of Cd4+and CD8+single positive cells was increased; this phenotype is reminiscent of thymic atrophy induced by stress inflammation. Further examination revealed that the natural killer T (NKT) (iNKT or NKT) cells expressed the transcription factor Ror γ T and generate interleukin-17 (IL-17) (Fig. 3D), which is characteristic of the NKT17 subgroup, one of the three subgroups of NKT cells in addition to NKT1 (which expresses T β) and NKT2 (expressing Gata3). In contrast, NKT cells from wild-type mice mainly consist of NKT1 and NKT2 subpopulations.

Methylation-induced gene expression differences eventually lead to the differentiation of immune cells and changes to the immune microenvironment of tumors, ultimately influencing the occurrence and development of tumors.

Antibody-dependent cell-mediated cytotoxicity (ADCC) occurs when the Fab segment of the antibody combines with the antigen epitope of virus-infected cells or tumor cells, and its Fc segment combines with the FcR on the surface of killer cells (e.g., NK cells, macrophages) to mediates the direct killing of target cells [95]. IgG antibody can induce these cells, primarily NK cells, to facilitate ADCC. In the process of antibody-mediated ADCC, the antibody can only specifically bind to the corresponding antigen epitope on the target cell, while the effector cells can kill any target cell that has been bound to the antibody; thus, the binding of the antibody to the antigen on the target cell is specific, while the killing effect of NK cells on the target cell is non-specific [96]. Methylation modification affects the differentiation of NK cells and determines the IgG antibody production of immune cells, thereby playing a decisive role in tumor immunity (Fig. 3E) [97].

Eukaryotes commonly use DNA methylation to inhibit the transcription of transposable elements that have been integrated into the host genome, such as endogenous retroviruses (ERV; Fig. 3F) [98]. Hypomethylated drugs stimulate natural antiviral response (so-called virus simulation) after promoting the re-expression of ERV in tumor cells. The two-way transcription of ERV produces double-stranded RNA (dsRNA) products. The dsRNA sensing pathway triggers a signal cascade involving the aggregation of mitochondrial antiviral signal proteins (MAVS), which ultimately passes through pro-inflammatory transcription factors (such as interferon regulatory factor 7 (IRF7) and nuclear factor-kappa-B (NF-κB)) to initiate an innate immune response to directly upregulate IFN type I α and IFN β (Fig. 3F) [99].

Autocrine and paracrine IFN α/β signal transduction in the tumor microenvironment can transmit the production of pro-inflammatory cytokines and chemokines, thus enhancing the immunogenicity of tumor cells (Fig. 3F) [100]. Specifically, hypomethylated drugs can be used to inhibit DNMT, which is sufficient to stimulate the transcription of ERV elements and induce a virus simulation state. The hypomethylation reagent induces bidirectional transcription of ERV elements (step 1) to produce dsRNA products (step 2), which are then exported to the cytoplasm and detected by innate immune mechanisms (step 3) [101]. After the detection of host pattern recognition receptors (e.g., melanoma differentiation associated protein 5 (MDA5)), the presence of cytoplasmic dsRNA triggers the signal cascade, and the combination of MDA5 and dsRNA induces the aggregation of mitochondrial antiviral signal proteins (MAVS) and the recruitment of TANK-binding kinase 1 (TBK1) (step 4). TBK1 phosphorylates IRF7, stimulates nuclear translocation and binding with normative binding motifs (step 5), and stimulates the transcription of interferon response genes (IRGs) (step 6) [102]. IRGs include type I/III IFNs, which are transcribed and translated before being transported to the cell surface and secreted into the tumor microenvironment (step 7) [103]. IFN secreted into the microenvironment is involved in activating the IFN signal pathway of adjacent cells (step 8) [104]. STAT1 protein is phosphorylated and modified by JAK1 to form heterologous dimers and recruit IRF9 (step 9). It is transferred into the nucleus and combined with the transcription factor IRSE to regulate the transcription activation of downstream target genes [105]. Viral mimics increase the immunogenicity of cancer cells and enhance antitumor immunity. Secreted type I/III interferons diffuse into the tumor microenvironment, resulting in effective autocrine and paracrine activation of the type I/III IFN pathway and inducing significant upregulation of genes involved in antigen presentation (step 10) [106]. The increased presentation of new tumor-derived antigens on APCs by MHC class I leads to increased cross-presentation of CD8+T cells (step 11). Therefore, activated tumor antigen-specific CD8+T cells can easily recognize new antigens through the homologous T cell receptor (TCR) and eliminate tumor cells by using the effector mechanism, including the production of inflammatory cytokines and cytotoxic particles (Step 12) [107].

### 2.4 Effect of histone modification on tumorigenesis and development

**Fig. 4:**
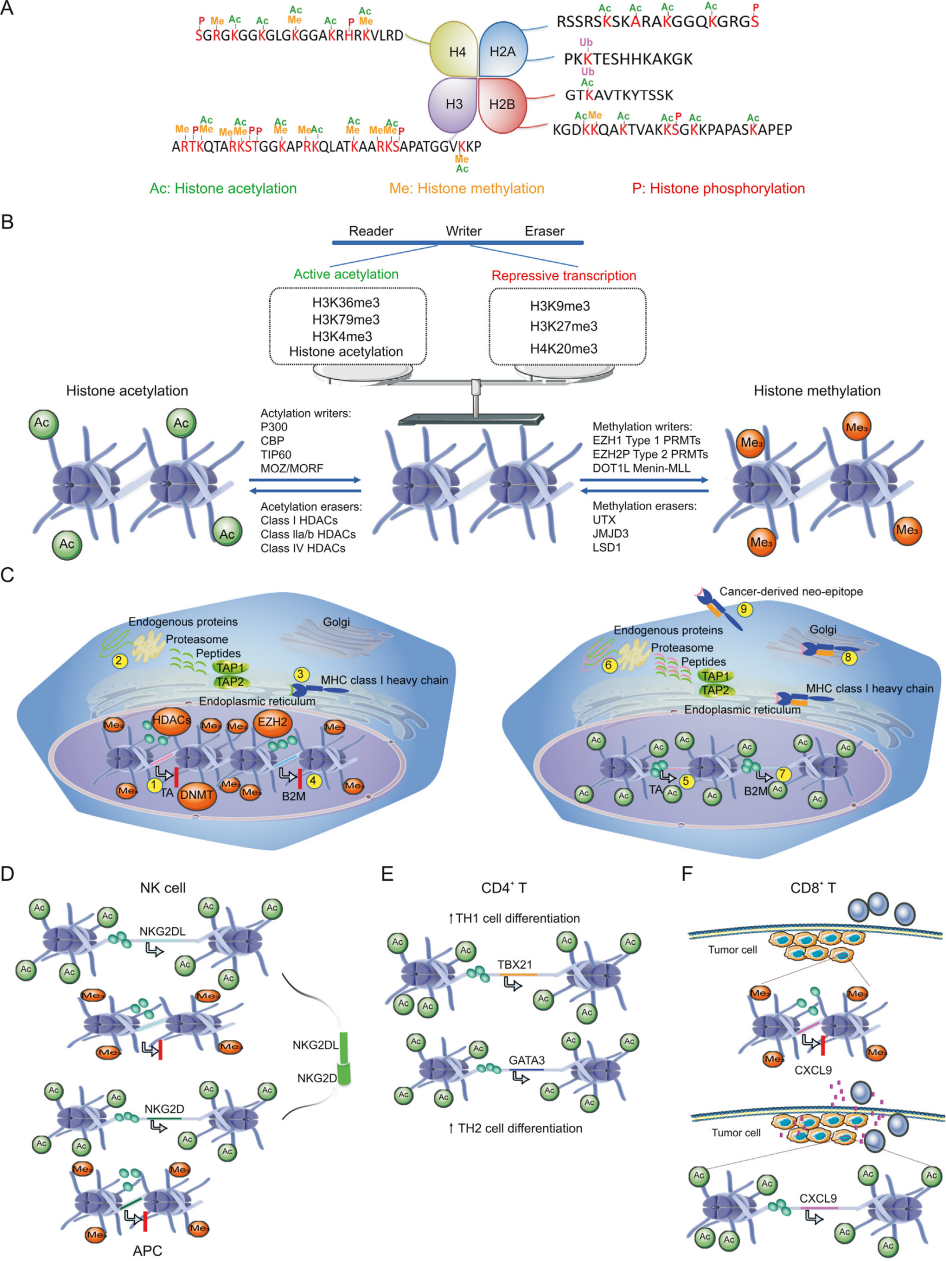
Effect of histone modification on the tumor immune microenvironment. (A) Histone H3 participates in the chromatin structure of eukaryotic cells alongside H2A, H2B, and H4. (B) Histone modifications include writer, eraser, and reader enzymes, all of which are personified terms. Writers refer to enzymes that modify histones, such as histone acetyltransferase (HAT). Erasers refer to unmodified transferases, such as histone deacetyltransferase (HDAC). “Readers” refer to enzymes that can specifically bind to certain modification markers. Various enzymes modified by histones participate in a wide range of activities and maintain balance in the body. The histone-modifying enzyme HDAC affects tumor immunity and ultimately regulates tumor formation and development by inducing the expression of major histocompatibility complex (MHC) class I antigen processing and presentation genes, including TAP1, TAP2, LMP2, LMP7, and B2M. (C) HDAC deacetylates histones, tightly binds to negatively charged DNA, tightly curls chromatin, and inhibits gene transcription. HDAC can inhibit the expression of MHC class I antigen processing and presentation genes, including TAP1, TAP2, LMP2, LMP7, and B2M, and downregulate immune checkpoint ligands, including PDL1. After using HDAC inhibitors, the chromatin structure becomes more open, promoting the expression of MHC class I antigen processing and presentation genes, and upregulating the immune checkpoint ligand PDL1. (D) Histone modification affects the recognition of natural killer (NK) cells by antigen presenting cell (APC) cells. (E) Histone modification affects the differentiation of CD4+T cells. (F) Histone modification regulates tumor cell release signals and induces the targeting of tumor cells by CD8+T cells. Me: histone methylation; Ac: histone acetylation; P: histone phosphorylation; Ub: histone ubiquitination; CBP: CREB binding protein; DOT1L: disruptor of telomeric silencing 1-like; EZH: enhancer of zeste homolog ; LSD1: lysinespecific demethylase 1.

Histone H3, with H2A, H2B, and H4, participates in the structure of chromatin in eukaryotic cells. Histone H3 participates in and affects many cell processes, including transcriptional activation/inactivation, chromosome packaging, and DNA damage/repair, through several types of epigenetic modifications, including acetylation, phosphorylation, methylation, ubiquitination, and ADP-ribosylation [108]. These modifications mainly occur in the N-terminal tail region of histone H3, which results in the rearrangement of the nucleosome structure into a more accessible open conformation transcription complex. In most species, histone H3 is acetylated at lysine 9, 14, 18, 23, and 56, methylated at lysine 4, 9, 27, 36, and 79, and phosphorylated at ser10, ser28, Thr3, and Thr11 (Fig. 4A) [109].

Post-translation acetylation of proteins is highly dynamic and regulated by the opposite activities of histone acetyltransferase (HAT) and HDAC (Fig. 4B) [110]. HDAC acetylation of histones is usually associated with chromatin coagulation and transcriptional silencing, and tends to be accompanied by an increase in histone methylation at the same residue [111]. HDACis induce acute hyperacetylation of histones and epigenetic regulators at the chromatin interface, resulting in transcriptional regulation driven by RNA polymerase II (Pol II) [112]. In cancer cells, this transcriptional regulation includes the re-expression of genes silenced during tumorigenesis, including tumor suppressor genes, antigen processing and pre-gene mechanisms, and tumor antigens [113]. A competitive histone acetylation-methylation balance is crucial for transcriptional regulation. Writers and erasers, modified by various histones, participate in the regulation and maintenance of the balance [114]. Histone is acetylated by various histone acetyltransferases [115]. Hyperacetylated histones are generally associated with an open chromatin structure that can be utilized by transcription factors and mechanisms. Histone acetylation is dynamic and depends on the net activity of HAT and HDACs at a given location [116]. In contrast, the kinetics of histone methylation are significantly slower, which makes this post-translational modification relatively stable. HMT can monomethylate, dimethylate, or trimethylate lysine and arginine residues in the histone tail, which can be removed by histone demethylase [117]. Highly methylated histone residues, such as K27 trimethylated histone H3 (H3K27me3), are associated with chromatin condensation and transcriptional silencing. It is worth noting that this simplified model of histone coding cannot reconcile the multiple modifications existing in a single nucleosome, nor can it reconcile the frequent contradictions of these rules. Indeed, histone markers control cell transcription in a cooperative, coordinated, and complex manner, rather than simply acting as an “off/on” switch. This study focuses on selected HATs, HDACs, HMTs, and histone demethylases and their small molecular inhibitors [118].

In cancer models, the efficacy of HDACis decreases in mice with low immune function or with the depletion of immune cells in wild-type mice, indicating that their mechanism of action possesses an immune-dependent component. Sodium valproate, a low affinity pan HDACi, can induce the expression of NKG2D ligands MICA, ULBP2, and ULBP3 in tumor cells, including primary patient acute myeloid leukemia (AML) cells, resulting in enhanced CD107a degranulation of NK cells. In a mouse melanoma model, the inhibition of HDAC class I with romidepsin increased the expression of MHC class I and decreased the cytolytic activity of CD8+T cells [119]. HDAC inhibition also induces the expression of MHC class I antigen processing and presentation genes, including TAP1, TAP2, LMP2, LMP7, and B2M (Fig. 4C), and up-regulates immune checkpoint ligands, including PDL1 [120]. The ability of tumor cells to escape recognition by the adaptive immune system can be mediated by the coordinated epigenetic silencing of tumor antigen (TA) and antigen presentation (AP) mechanisms [121]. TA may be silenced in cancer cells through polycomb inhibition of complex 2 (PRC2) activity (and inhibition of H3K27me3 deposition), high HDAC activity (destruction of activated histone acetylation markers), and DNMT-mediated methylation (transcriptional inhibition TA) (step 1). Endogenous cellular proteins are degraded by the proteome, and the generated peptides are introduced into the endoplasmic reticulum by transporters related to antigen presentation (TAP) proteins TAP1 and TAP2 (step 2). In the endoplasmic reticulum, peptides are loaded onto the MHC class I complex, which includes the MHC class I heavy chain, and β 2-microglobulin (β2M) is then transported to the cell surface (step 3) [122]. In addition to TA, various components of the AP mechanism can be transcribed and silenced through the above epigenetic mechanism to promote immune escape (step 4). In this example, epigenetic silencing of the β2M locus prevents MHC class I from being transported to the cell surface, thus limiting the recognition of antigens and new antigens. Targeted epigenetic regulatory factors reduce the transcriptional inhibition of TA and AP mechanisms to re-participate in antitumor immunity. Epigenetic therapy targeting PRC2, HDAC, or DNMT can activate TA transcription (step 5). After translation, TA enters the proteasome pathway and facilitates transport of the new antigen peptide and traditional endogenous peptide to the endoplasmic reticulum through TAP1/TAP2 (step 6). Active transcription of all necessary AP mechanisms leads to the formation of a peptide-MHC class I complex, including TA-derived peptide (in this case, β2M stable peptide-MHC class I complex to allow trafficking) (step 7) [123]. Then, the TA–MHC class I complex is routed to the cell surface through the Golgi apparatus (step 8). Finally, MHC class I presents a new immunogenic epitope to cytotoxic CD8+lymphocytes (step 9) (Fig. 4C) [124].

Regarding the epigenetic regulation of NK cells, histone acetylation enhances the expression of NKG2D on NK cells and NKG2DL on tumor APCs, which leads to NK cell activation and tumor cell death [125]. EZH2 inhibition can also increase the expression of MHC class I, which is a negative regulator of NK cell function. The expression of NK cell effector genes such as *IFNG* is regulated by DNA methylation, so DNMT inhibitor (DNMTi) may be used to enhance the expression of these genes, thus improving the cytotoxicity of NK cells. In terminally differentiated NK cells, the TSS of key effector genes, including *IFNG* and *TBX21* (encoding T-Bet), is characterized by DNA hypomethylation and extensive histone acetylation. Therefore, after being stimulated by cytokines or NK cell ligands expressed on tumor cells, the terminally differentiated NK cells are epigenetically pretreated for powerful pro-inflammatory and cytotoxic reactions [126]. Recently, the results of *in vitro* compound screening showed that inhibition of histone demethylase JMJD3/UTX with GSK-J4 reduced the expression of several pro-inflammatory cytokines, including IFN γ, TNF, and granulocyte-macrophage colony-stimulating factor (GM-CSF). Consistent with this effect, inhibition of the trimethylation of H3K27 with EZH2is, and inhibition of histone deacetylation with inhibitors of HDAC type I/II/IV resulted in increased gene expression related to NK cytotoxic function, including Klrk1 (encoding NKG2D), and increased cytotoxic activity *in vitro*. Small molecule EZH2 inhibition can simultaneously up-regulate NKG2D ligand on tumor cells, indicating that EZH2i can enhance NK killing by simultaneously regulating important NK activation molecules on NK cells and tumor cells (Fig. 4D).

Regarding epigenetic regulation of CD4+T cells, EZH2 deposits inhibitory chromatin markers that limit the expression of major transcription factors of T-helper cells that determine cell lineages; therefore, inhibition of EZH2 results in the increased differentiation of TH1 and TH2 cells. SET domain bifurcation 1 (SETDB1) limits the expression of TH1 cell-related genes such as *TBX21*, and inhibiting this complex can enhance the TH1 cell response and promote antitumor immunity (Fig. 4E) [127].

The limited infiltration of T cells into the tumor microenvironment may be downstream of the epigenetic silencing of key chemokines such as CXCL9 and CXCL10, which encompasses inhibitory histone modification and DNA methylation. Targeted inhibition of DNMT, EZH2, HDACs, and lysine-specific demethylase 1 (LSD1) has been shown to induce the expression of chemokines resulting in increased T cell infiltration into the tumor microenvironment and enhanced antitumor immunity (Fig. 4F).

In the context of cancer, NK cells treated with 5-Aza show enhanced effector function, highlighting the further potential of epigenetic manipulation. Additionally, treatment of mice with trastuzumab and inhibition of I/II/IV HDAC by Penobinast promoted the NK-mediated response to HER2+ (also known as ERBB2+) breast adenocarcinoma. The mechanism of this reaction is indirect through the enhanced expression of CXCL9/CXCL10, and results in increased NK cell recruitment, rather than direct regulation of the cytotoxic function of NK cells.

### 2.5 Effect of the 3D structure of chromatin on tumorigenesis and development

The change in gene expression is mainly regulated by DNA-binding transcription factors. Intercellular communication and microenvironment sensing trigger an intracellular signal cascade, which terminate with transcription factors activation, allowing the tissue microenvironment to alter gene transcription and cell phenotypes. Transcription control involves many regulatory elements, including promoters upstream of gene transcription initiation sites and enhancers, which control transcription at large genomic distances. DNA can be methylated, which can attract or repel specific transcription factors and regulatory proteins. Accessibility of DNA to transcription factors is controlled by the localization and post-translational modification of histones that package DNA into chromatin [128]. DNA methylation or histone modification ultimately alters chromatin structure, affects the formation of the enhancer–promoter ring, and changes the expression of downstream genes [129]. In this part, we discuss the latest insights on how 3D genome folding affects the gene regulation process to form the differentiation, activation, dysfunction, or malignant transformation of immune cells [130].

**Fig. 5:**
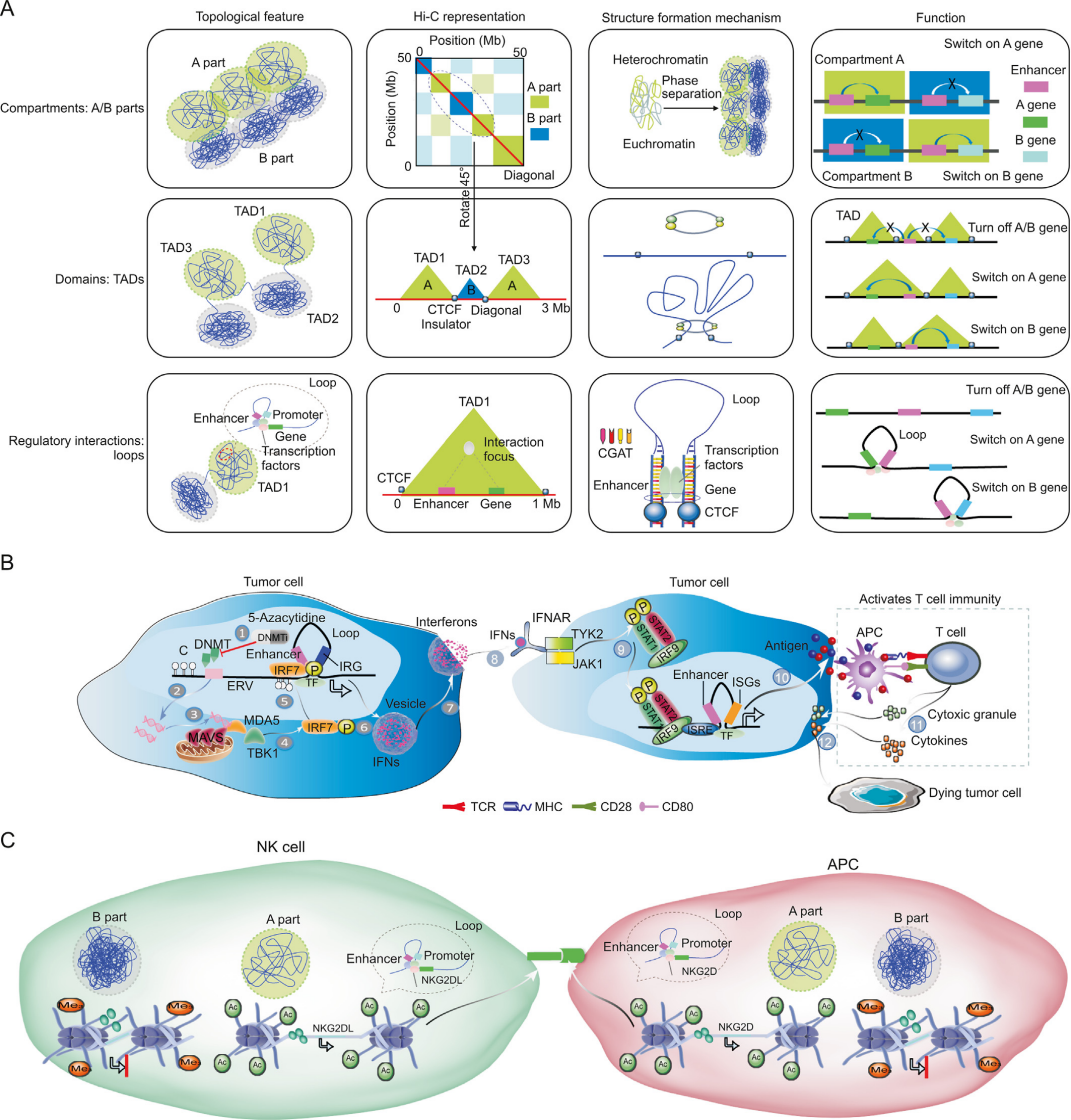
Effect of the 3D structure of chromatin on the tumor immune microenvironment. (A) The 3D structure of chromatin is involved in regulating several important biological processes, such as DNA replication and gene expression, and is closely related to the occurrence and development of diseases. The advanced structures of chromatin are divided into a chromatin loop, topologically associated domain (TAD), and A/B compartment. (B) Phosphorylated interferon regulatory factor 7 (IRF7) acts as a transcription factor, enriches other transcription factors, forms an enhancer–promoter loop, and initiates the transcription of interferon response gene (IRG). The polymer–protein complex combines with the interferon-stimulated response elements (ISRE) to form an enhancer–promoter loop, which begins the transcription of *ISG* genes. (C) DNA methylation and histone acetylation lead to chromatin structure and gene expression changes in natural killer (NK) and antigen presenting cell (APC) cells, which finally determine the recognition of APC cells by NK cells. CTCF: CCCTC-binding factor; IFNs: interferons; IFNAR: interferon alpha receptor; TCR: T cell receptor; MHC: major histocompatibility complex.

Three main tissue layers define the 3D structure of chromatin in interphase nuclei [131]. Active and inactive chromatin (A part/B part), defined by transcriptional activity, histone modification, and DNA accessibility, divides the genome into two main clusters of self-interaction domains, termed A and B regions (active and inactive chromatin, respectively). These compartments generate a characteristic chessboard representation in the Hi-C contact diagram. The gene in the A part region has a relatively loose chromatin structure, which is usually accompanied by acetylation of chromatin. The promoter region can combine with transcription factors to promote gene expression. At the sub-giant scale, the genome is further divided into self-interaction chromatin domains called TADs, which are shown as triangles with increased interaction on the horizontally inclined Hi-C diagram. At a finer resolution, specific contacts in TAD (which can be seen as dot signals in the Hi-C diagram) recognize the interaction between genes and remote regulatory elements. Generally speaking, the TAD is an isolated region, and the promoter and enhancer in a TAD are more likely to fold close [132]. In terms of the mechanism, phase separation is considered a biochemical force to promote zoning [133], while the annular extrusion of the viscous complex and CTCF are considered as the main driving factor for the formation of TAD [134]. Collaboration between DNA-binding transcription factors (i.e., enhancers and promoters) to establish a regulatory interaction between the enhancer and promoter, which is believed to involve the formation of a specific transcriptional condensate through local phase separation [135]. In terms of function, separation may be necessary to ensure the stability of transcription and prevent the mixed de-expression of heterochromatic regions, as well to promote the faster activation of promoter genes (for example, if they are already located in region A) [136]. The TAD boundary concentrates the interaction between the regulatory regions in the same TAD and inhibits the interaction with the adjacent TAD regions in a process that is essential for promoting correct enhancer–promoter interaction and fine-tuning transcription dynamics (Fig. 5A) [137].

### 2.6 Genomic topology in the development of immune cells

Regarding 3D genomic tissue of hematopoietic stem and progenitor cells, the hematopoietic system provides an attractive model for exploring the gene regulation mechanism controlling cell differentiation [138]. Hematopoietic stem cells (HSCs) produce billions of mature blood cells every day, including all major immune cell types, in a process that requires precise and coordinated regulation of thousands of genes to respond to the prompts of the microenvironment (Fig. 2) [139]. Recent research has begun to explore the 3D genome organization under the background of the fate differentiation of immune cells [130].

Before birth, fetal liver HSCs migrate to the bone marrow where they remain until adulthood. Due to different transcription schemes, these HSC subtypes show different proliferative capacities and differentiation output [140]. In mice, the genomic topological structure is essentially conserved between fetal liver HSCs and adult bone marrow hematopoietic stem cells, although the latter shows a more robust psychological and TAD boundary strength [141]. However, stage-specific enhancer–promoter interactions are widespread and are associated with the gene regulation of phenotypic differences between fetal liver and adult bone marrow HSCs (including HSCs involved in cell cycle control) [142]. HSCs differentiate into immune cells through the sequential differentiation of several progenitor cells. Interestingly, large regions lacking DNA methylation have been shown to form specific large base-level chromatin rings in mouse primary stem cells and progenitor cells, in a process that was independent of ring extrusion 45 [143]. These DNA methylation “canyons” are rich in trimethylated histone H3K27 (H3K27me3), which is required for spatial clustering and stem cell identity gene transcription control 45 [144]. Hi-C analysis showed that during mouse HSC differentiation, the dynamic rearrangement of chromatin compartments showed that the overall increase of chromosome aggregation was accompanied by the appearance of Rabl configuration, in which the centromere was at pole 46 of the nucleus [145]. The support for the link between genome condensation and maturation of immune cells in chamber B comes from the loss of histone H1 in mouse hematopoietic cells *in vivo*, which significantly reduces the number of mature lymphocytes, chromatin densification, and B cell maturation 47 [146]. Indeed, differentiation into neutrophil progenitor cells induces a strong long-term interaction between transcriptional silencing regions, resulting in chromatin densification [147]. Interestingly, ZNF143 TF binding controls local CTCF chromatin recessions in hematopoietic progenitor cells, allowing the ZNF143–CTCF-mediated enhancer–promoter loop to regulate stem cell maintenance of gene expression. On a smaller scale, strong and uniform interaction domains that overlap with the genome (genome-related domains) appear in different hematopoietic cells, which are independent of the formation of CTCF [148]. Genome-related domains have been found at highly expressed genes and are dynamically correlated with gene expression during hematopoietic cell differentiation.

Regarding the effect of methylation on tumor immunity outlined in Fig. 3, it is the dsRNA sensing pathway that triggers the signal cascade involving the aggregation of mitochondrial antiviral signal proteins (MAVS), before proinflammatory transcription factors (such as IRF7 and NF-κB) initiate the innate immune response [99]. Tumor microenvironment α/β autocrine and paracrine IFN in signal transduction can transmit the production of pro-inflammatory cytokines and chemokines, thus enhancing the immunogenicity of tumor cells (Fig. 3F) [149]. Hypomethylation drugs can be employed to inhibit DNMT, which is sufficient to stimulate the transcription of ERV elements and induce the virus simulation state [150]. Here, DNA methylation is inhibited to make chromatin having more space for transcription activities (step 1) [151]. TBK1-phosphorylated IRF7 is stimulated and translocated in a hypomethylation environment. Here, phosphorylated IRF7 acts as a transcription factor, enriches other transcription factors, forms an enhancer–promoter loop, and initiates the transcription of IRG [152]. STAT1 protein is phosphorylated and modified by its kinase JAK1 to form a heterologous or homodimer and recruit IRF9 [153]. This protein polymer acts as a transcription factor and combines with other transcription factors such as IRSE (Step 9) [154]. The polymer–protein complex combines with the transcription factor IRSE to form the enhancer–promoter loop, which initiates the transcription of *ISG* genes. This example illustrates that DNA methylation is closely related to the change in chromatin structure and ultimately determines the change in the tumor immune microenvironment (Fig. 5B) [155,156]. Histone modification regulates NK cells by causing the chromatin structure to become a loose A part region (Fig. 4D) [157]. The regulation region of NKG2D has more space to combine with related transcription factors to form an enhancer–promoter ring of NKG2D, thus promoting NKG2D expression. The same is true of tumor APCs, which initiates the expression of NKG2DL, leading to NK cell activation and tumor cell death (Fig. 5C) [158]. This example illustrates that histone modification is closely related to the change in the chromatin structure. Either histone modification or DNA methylation will eventually change the structure of chromatin, resulting in altered expression of genes, determining tumor formation or restriction [159].

## 3 Methods for studying the epigenetic modification

Epigenetic modifications affect the growth and development of organisms, disease progression, etc. Therefore, a large number of research methods have been developed to detect epigenetic modifications, including DNA methylation, histone modifications, chromatin structure, etc (Table 2) [160]. DNA methylation is one of the earliest discovered epigenetic modifications of genes. It plays an important role in maintaining normal cell function, genetic imprinting, embryonic development and tumorigenesis, and is both a current and future research hotspot. With the development of high-throughput sequencing technology, we can analyze events such as 5′-methylcytosine and histone modification at the whole-genome level using “DNA methylation sequencing” to provide more in-depth results than those obtainable by genomics research [161]. Moreover, with the continuous decline of sequencing costs and the iterative updating of sequencing technology in recent years, DNA methylation sequencing methods can be more selective [162]. At present, there are six common sequencing methods for epigenetic DNA methylation research: whole-genome bisulfite methylation sequencing (WGBS), precise DNA methylation and hydroxymethylation sequencing (oxidation combined with bisulfite transformation sequencing (oxBS-Seq)), optimized simplified methylation sequencing (RRBS/dRRBS/XRS), single/micro cell whole-genome methylation sequencing (scWGBS), amplification (hydroxy) methylation sequencing, and (hydroxy) methylated DNA immunoprecipitation sequencing ((h) MeDIP-seq), with each solution suitable for different DNA methylation research directions [163].

**Table 2:** Methods used in epigenetic research.

Research content	Research method	Experimental principles
Chromatin accessibility	MNase-seq	Mnase enzymatic digestion of DNA fragments not occupied by nucleosomes or transcription factors.
	DNase-seq	Cutting open chromatin regions using DNase I.
	FAIRE-seq	Using ultrasound to fragment DNA and separating DNA fragments with phenol chloroform.
	ATAC-seq	Cut open chromatin regions using Tn5 enzyme and insert labeled fragments.
Histone modification	Chip-seq	Using ultrasound to fragment DNA and separating DNA fragments with phenol chloroform.
	Cut-tag	Cut open chromatin regions using Tn5 enzyme and insert labeled fragments.
DNA methylation	WGBS	The product is transformed from unmethylated cytosine C to uracil U through bisulfite treatment, and then from uracil U to thymine T through linker sequence mediated PCR technology.
	oxBS-seq	5hmC is oxidized by KRuO4 to 5fC, and after being treated with bisulfite, it is deammoniated and read as T, while 5mC is still read as C. Therefore, 5mC and 5hmC can be distinguished at the single base level.
	MeDIP-seq	Enrichment of DNA methylation regions in the genome using 5mC antibodies and high-throughput sequencing were performed to detect methylation sites in high CpG regions of the genome.

MNase: micrococcal nuclease; Seq: sequencing; FAIRE: formaldehyde-assisted isolation of regulatory elements; ATAC: assay for transposase-accessible chromatin; WGBS: whole genome bisulfite sequencing; oxBS: oxidative bisulfite; MeDIP: methylated DNA immunoprecipitation; 5fC: 5-formylcytosine; 5mC: 5-methylcytosine; 5hmC: 5-hydroxymethylcytosine.

**Fig. 6:**
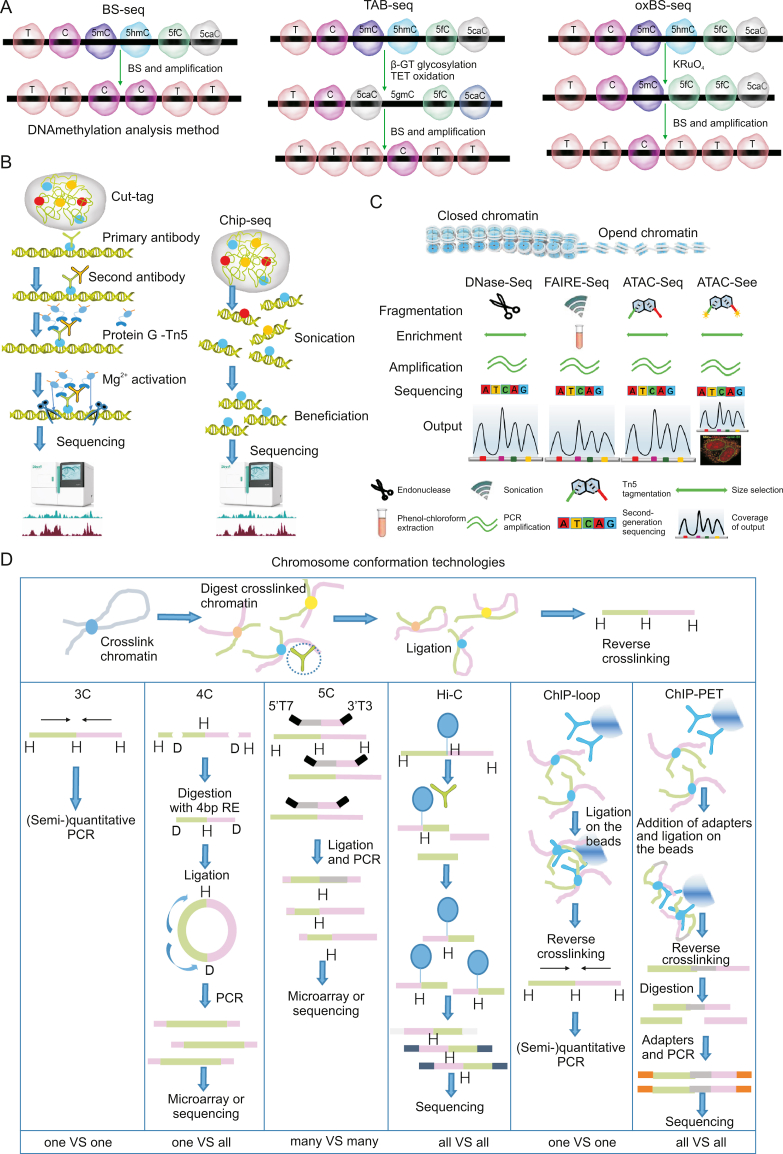
Methods for studying epigenetic modification. (A) The principle of methylation sequencing. The main principle is that treating DNA with bisulfite can convert cytosine residue (C) into uracil (U), but 5-methylcytosine residue (5mC) is resistant to it and does not undergo transformation. Therefore, bisulfite treatment introduces specific changes in the DNA sequence that depend on the methylation status of a single C residue, thus generating single nucleotide resolution information about the methylation status of DNA fragments. Whole genome bisulfite sequencing (WGBS) is widely used in methylation sequencing, mainly detecting two modification forms: 5mC and 5-hydroxymethylcytosine (5hmC). Based on this, some methods have been derived to distinguish between 5mC and 5hmC, such as oxidative bisulphate-seq (oxBS-seq) for 5mC-specific sequencing and ten-eleven-translocation (TET)-assisted TET-assisted bisulphate-seq (TAB-seq) for 5hmC-specific detection. (B) Chip-seq and cleavage under targetsand tagmentation (Cut-tag) are used to investigate the interaction between specific proteins and DNA. Cut-tag is a more efficient experimental design proposed in recent years, which uses Tn5 enzyme to cut DNA fragments, reducing the number of cells required for the experiment. (C) Methods have been developed to explore the chromatin accessibility. DNase seq used restriction endonuclease (DNase I) to fragment the sample. In the dense regions of chromatin, DNA strands are well protected by dense structures, making it difficult for endonucleases to access these regions and only capable of cutting open regions of DNA. Formaldehyde-assisted isolation of regulatory elements (FAIRE)-seq is an alternative approach to identify genomic regulatory regions. Assay for transposase-accessible chromatin with high-throughput sequencing (ATAC-seq) uses a modified Tn5 transposase to design transposable DNA as a junction and randomly insert it into the open region of chromatin. ATAC-see combine both imaging and sequencing technologies to reveal the accessible genome. (D) Chromosome conformation capture (3C) technology mainly studies point-to-point gene interactions, that is, the interaction between specific two genes, such as the interaction between gene A and gene B. Studying the interaction between a gene and multiple genes, or between multiple genes and multiple genes, requires the use of chromosome conformation capture-on-chip (4C), chromosome conformation capture carbon copy (5C), high-through chromosome conformation capture (Hi-C) techniques. Exploring gene interactions under specific proteins requires ChIP-loop and chromatin interaction analysis using paired end tag sequencing (ChIP-PET) techniques. BS-seq: bisulfite-seq;

WGBS can accurately detect the methylation level of all single cytosine bases (C bases) in the whole genome, and is the gold standard for DNA methylation research [164]. WGBS can provide important technical support for the research of spatiotemporal specific modification of genomic DNA methylation, and can be widely used in the mechanism research of life processes such as individual development, aging and disease, as well as being the preferred method for the study of methylation maps of various species. Conventional whole-genome methylation sequencing technology uses T4-DNA ligase to connect the connector sequence at both ends of the genomic DNA segment interrupted by ultrasound [165]. The connector product converts the unmethylated cytosine C into uracil U through bisulfite treatment, before converting uracil U into thymine T through polymerase chain reaction (PCR) technology mediated by the connector sequence [166]. Technical advantages of WGBS include its wide application range, applicable to human and most animal and plant research (reference genome is known), whole genome coverage, maximum access to complete genome methylation information, and the ability to accurately draw a methylation map. Additionally, the single base resolution can accurately analyze the methylation status of each C base [167]. DNA hydroxymethylation is a new DNA modification discovered in recent years and has rapidly become a research hotspot. Crucially, with the deepening of research, it was found that bisulfite sequencing, previously considered as the “gold standard” for detecting DNA methylation, could not distinguish DNA methylation (5mC) from DNA hydroxymethylation (5hmC) [168]. Chemical oxBS-Seq can accurately detect not only DNA methylation and eliminate the influence of DNA hydroxymethylation but also DNA hydroxymethylation, by simultaneously combining double libraries with single base resolution [169]. The technical principle of DNA hydroxymethylation can be summarized as follows: oxBS-Seq oxidizes 5hmC to 5fC, which can be converted into U by bisulfite to achieve accurate detection of 5mC; simultaneously, the accurate detection of 5hmC can be achieved by comparing with the conventional bisulfite results. scWGBS involves the study of DNA methylation genomics of single cells and microsamples is largely subject to the construction of database sequencing technology [170]. Traditional library construction methods or single-cell amplification techniques similar to genomic DNA are difficult to apply to methylation experiments. The new experimental method establishes a technology based on linear amplification and single-tube library construction, which can fully reduce the library preference and accurately complete the whole-genome methylation study of precious samples [171](Fig. 6A).

Chromatin immunoprecipitation followed by sequencing (ChIP-Seq) is a method used to explore the histone modification region, the transcription factor binding state, and the DNA-protein interaction in the whole genome [172]. Because the steps of formaldehyde cross-linking, chromatin fragmentation, immunoprecipitation, and library construction in ChIP-Seq are cumbersome and difficult to control, and conventional ChIP-Seq requires a large number of cells, with poor repeatability and a low signal-to-noise ratio [173]. These technical difficulties limit the further application of ChIP-Seq in DPI research and hinder the development of epigenome and other fields. Cleavage under targets and tagmentation (CUT&Tag) technology is a new DNA-protein interaction research method, which is expected to replace the traditional ChIP-seq technology to explore histone modification regions, transcription factor binding status, and DNA-protein interaction in the whole genome [174]. CUT&Tag is favored by epigenetic researchers because of its advantages such as simple operation (without immune co-precipitation and ultrasonic fragmentation), low initial cell volume (as low as 60 cells), high signal-to-noise ratio, high sensitivity, good repeatability and low cost [175]. The core of CUT&Tag technology is the transposon pAG-Tn5, which is fused with the protein A/G antibody binding domain on Tn5 [176]. In the CUT&Tag experiment, the target protein specific antibody (the first antibody) is incubated first to cause the antibody to enter the cell and bind to the target protein. To amplify the signal, the second antibody is incubated in the same way [177]. Finally, the pAG-Tn5 transposon was incubated to make the transposon enter the cell and bind with the antibody so that the transposon is indirectly fixed on the target protein, and then Mg^2+^ is added to activate the cutting activity of Tn5 enzyme and interrupt the DNA region of the target protein binding [178]. As Tn5 is connected with a sequencing connector, the connector is directly added to the fragmented DNA at the same time of interruption, and finally, the DNA is extracted for PCR amplification to build the library (Fig. 6B) [179].

As outlined above, not only DNA methylation and histone modification but also the spatial structure of chromatin affect gene expression. The binding of open regions on chromosomes with corresponding transcription factors or other regulatory proteins directly affects the occurrence of gene replication and transcription in cells [180]. Accurate identification of these specific open DNA regions on the genome is critical to the discovery of genomic regulatory elements [181]. The DNA sequence without nucleosome protection has higher activity than the DNA sequence wrapped on the nucleosome, and is easier to be cut or interrupted by nuclease, transposase, or physical and chemical means to form DNA fragments of different lengths [182]. Therefore, current research on chromatin accessibility mainly uses enzymolysis or ultrasonic treatment to segment DNA in the open region. At present, four main methods are employed to study the accessibility of chromatin: MNase-seq, DNase-seq, FAIRE-seq, and ATAC-seq [183]. MNase-seq indirectly reflects the accessibility of chromatin by sequencing the DNA protected by nucleosomes, while the other three methods directly reflect the accessibility of chromatin by detecting the open region on chromatin [184].

In the MNase-seq database building experiment, the chromatin of the cell is fixed with formaldehyde in advance, and then treated with excessive MNase to obtain the DNA wrapped on the histone of a single nucleosome, before finally conducting second generation sequencing analysis [185]. The standard MNase-seq is mainly used for sequencing nucleosome fragments (∼147bp), which limits the analysis of non-histone proteins outside the nucleosome at the DNA-binding sites [186]. In general, MNase-seq is an excellent method for detecting the distribution of whole-genome nucleosomes and evaluating the binding of transcription factors, which can be used in many types of cells. However, to obtain better repeatability and comparability in different experiments, MNase-seq needs many cells and strict enzymatic hydrolysis conditions [186]. DNase-seq deoxyribonuclease I (DNase I), an endonuclease encoded by the human gene DNASE1, can cut double-stranded DNA non-specific. DNase I sensitive sites are considered to have the characteristics of open and accessible chromatin in genomics and chromatin research [187]. The low concentrations of DNase I can cut open regions occupied by non-nucleosomes in the genome, termed DNase I sensitive sites. The traditional method for identifying DHSs is mainly southern blotting of terminal markers, which involves many time-consuming and laborious steps [188]. The emergence of second-generation sequencing technology makes it possible to efficiently and specifically identify DHSs in the whole genome [189]. In the ENCODE alliance, DNase is widely used in the analysis of cell-specific chromatin accessibility and the study of the relationship between cell chromatin accessibility and gene expression [190]. The binding of transcription factors in DHSs prevent DNase from cutting DNA, so that the occupation of transcription factors can be observed at the single base level. Because DNase I has a certain preference when cutting DNA, the reliability of DNase-seq used for transcription factor imprinting detection has been questioned. Additionally, the operation of the experiment requires multistep sample preparation and enzyme titrations, and the concentration of DNase also needs to be adjusted for different cell types or cell dosages [191].

ATAC-seq is a method for detecting open chromatin developed by William Greenleaf from Stanford University in the United States in 2013 [192], which largely depends on the high sensitivity of Tn5 transposase to fragmented DNA and its integration into activated regulatory regions. The transposon is essentially a movable DNA segment, which can be moved in the genome without the help of DNA homologous series [193]. Bacterial transposons can be divided into different types, such as insertion sequences, transferable phage complex transposons, and the TnA transposon family [194]. Tn5 is a bacterial transposon that was first found in *E. coli*. Tn5 is a DNA segment containing several resistance and transposase genes belonging to a compound transposon. The Tn5 sequence is 5,818 bp long and consists of the core sequence encoding bleomycin, streptomycin, and neomycin and two inverted IS50 sequences that have high homology [195]. The IS50 contained two inverted ends, the outer end OE and the inner end IE of 19 bp. This inverted terminal is the action site of transposase, which plays an important role in the process of transposition. The right insertion sequence IS50R can encode a 53-kDa Tn5 transposase and can also encode a 48-kDa transposable repressor protein Inh. The Tn5 transposon is the transposon of the IS4 family [196]. The IS4 family is divided into several subgroups, in which the subgroup of IS50 contains 19 transposon elements [197]. Tn5 transposase recognizes the mosaic end (ME) sequence of 19 bp on both sides of the DNA segment. When the transposition event occurs, two transposase (Tnp) molecules bind to the OE end of the Tn5 transposon to form two Tnp-OE complexes, and then the two complexes interact through the C-end of the Tnp to form the Tn5 transposon complex. At this time, Tnp produces DNA cutting activity [198]. In ATAC-seq, 500–50,000 unfixed nuclei can be sequenced by Tn5 transposase. Due to the steric hindrance effect of nucleosomes, the Tn5 transposase carrying sequencing connector is mainly inserted into the open region integrated into the chromatin [199]. After PCR amplification, two-terminal second-generation sequencing is performed. The ATAC-seq database building process is simple and fast, requires a few cells, and can interpret the chromatin structure at a high resolution. Additionally, the database construction process does not include any fragment length screening and can simultaneously detect open DNA regions and regions occupied by nucleosomes (Fig. 6C) [200,201]. Howard Chang of Stanford University in the United States developed the ATAC-see technology, which uses the same enzymatic method as ATAC-seq to integrate DNA markers. The difference is that the fluorophore coupled with these DNA markers allows them to visualize the 3D fixed nucleus.

The key points of chromatin conformation capture (3C) technology can be summarized as follows: formalin fixes the nuclear chromatin instantaneously; excessive restriction endonuclease is used to digest the chromatin–protein crosslink; ligase is used to connect the digest under the condition of extremely low DNA concentration and extremely high ligase concentration; and albumen K digests the crosslink to release the bound protein. Primers that might interact with the target segments are used to conduct general PCR and quantitative PCR to determine whether there is interaction [202]. Moreover, 3C technology assumes that the connection frequency of physically interacting DNA segments is the highest, uses site-specific PCR to detect the physical contact between DNA segments in the genome, and finally determines whether there is interaction based on the abundance of PCR products [202,203]. The use of 3C technology is suitable for studying the interaction between 5 kb and hundreds of kb chromatin [204]. When conducting 3C experiments, the experimenter requires partial information about the DNA fragments being studied and the DNA fragments assumed to interact with them, in order to design PCR primers for PCR experiments and determine whether there is interaction between these fragments. However, this assumption has some limitations; in theory, there is no deviation in the estimation, and 3C technology can only explore one-to-one relationship [202]. Four-C (4C) technology, also known as circular chromatin conformation capture or chromatin conformation capture-on-chip, is based on 3C technology and can make up for this deficiency [205]. In 4-C technology, known DNA fragments (bait) and unknown DNA fragments (usually located in the gene regulatory region) have been enzymatically linked into a ring. Reverse PCR is carried out using bait-specific PCR primers. The sequence obtained from the PCR products can be considered to be the contact site between two distal sequences [206]. Sequence analysis of PCR products can determine the location of the interacting chromatin and the possibility of interaction [207,208]. To study the possible interaction between hundreds of chromatin fragments, using 3C technology requires designing several PCR primers to determine the relationship between known fragments and hypothetical fragments, which is low in flux and difficult to achieve [203]. Therefore, people have designed 3C carbon copy (5C) technology, which is based on the basic principle of 3C, combine ligation-mediated amplification (LMA) to increase the flux of 3C detection [209]. Take the 3C restriction enzyme library as a template and add universal connectors (such as T7, T3) at the 3C primer end, such as T7 connector at the 5′ end of the forward primer (bait) and T3 connector at the 3′ end of the reverse primer [210]. If two putative fragments are interconnected, due to the nature of ligase-mediated binding, only the connected fragments can be amplified. In this way, the general primers T7 and T3 can be used for PCR, and then the products can be sequenced with high throughput to achieve the high-throughput 3C experiment [209]. At present, professional 5C auxiliary websites can be used to help with the primer design for 5C experiments; indeed, 5C is actually a multiple 3C, which requires the design of many primers and cannot conduct whole-genome screening [[211], [212], [213]].

Hi-C technology is also developed based on the 3C principal and comprises the following main steps: cross-linking the chromosomes interacting with proteins in cells with formalin, restriction enzyme digestion, and then flattening the gap (dCTP for biotin labeling) [214,215]. Ligases are used to connect the sample and ultrasonic fragmentation is performed. Subsequently, fragments are precipitated using biotin affinity chromatography, and splices are added for deep sequencing. Then, a 3D spatial structure map of adjacent chromatin is constructed by splicing massive data. The difficulty in conducting Hi-C experiments is not in capturing the conformation of chromatin but in how to analyze and process the resulting massive data [[216], [217], [218]]. Chromatin itself exists in aggregation, so there is significant background noise in the obtained data; thus, how to eliminate background noise is a major difficulty in the application of Hi-C technology [219].

In living cells, a single DNA fragment mediated by protein factors may interact with multiple sites [220,221]. In different cells, the same DNA fragment may also interact with different sites, which may determine the temporal and spatial differences in gene expression in different cells [222,223]. The ChIP loop experiment [15] was developed based on 3C technology to study the interaction between candidate protein factors and target DNA fragments, commonly used are the ChIP-3C and ChIP-4C techniques [224,225]. In the experimental process, only after excessive restriction endonucleases digest the chromatin protein conjugate is immunoprecipitation is performed with the antibody specific to the protein factor under study, before finally performing the enzymatic product connection [[226], [227], [228]]. The subsequent steps are the same as the corresponding 3C and 4C steps. Notably, proteins precipitated using specific antibodies may act on sites adjacent to the target DNA rather than on interactions between the target DNA and other DNA [[229], [230], [231]]. It has been reported that blocking the expression of this protein factor can be used to determine whether the interaction between the target DNA fragment and other DNA truly exists [232].

Chromatin interaction analysis with paired-end Tag (ChIA-PET) technology is a combination of 3C paired end PET [18] and next-generation sequencing technology [233,234]. ChIA-PET can not only detect intracellular chromatin interactions but also solve problems such as small DNA fragments and maximum data obtained from experiments. ChIA-PET can unbiasedly identify chromatin fragments that interact with target protein factors across the entire genome [[233], [234], [235]]. Part of the experimental process is similar to the ChIP-loop experiment and the process can be summarized as follows: cells are immobilized with formalin; the genome is digested with restriction enzymes; the protein DNA complex is precipitated with antibodies specific to the target protein; a biotin labeled splice is added to the digested fragment (this splice has a specific cleavage site; such as MmeI); and a secondary ligation reaction is performed [224,236]. Then, an enzyme with a splice is used for digestion (such as MmeI) and the resulting product is coupled with a splice, before performing in-depth sequencing [[237], [238], [239]]. ChIA-PET can be used to identify the sites where the target protein interacts with DNA and further identify the genes that the target protein may regulate (Fig. 1) Understanding the spatial folding rules of chromatin is important for understanding the chromatin structure, gene activity, and cell differentiation (Fig. 6D) [240,241].

## 4 Conclusion and future perspective

Epigenetic modulators may synergistically affect tumor cells and non-tumor cells within the tumor microenvironment in a manner that facilitates immune recognition of tumor cells to support improved immune-mediated treatment outcomes. Under appropriate conditions, these drugs can promote tumor ICD and inhibit tumor angiogenesis and hypoxia within the tumor microenvironment, thereby improving immune cell infiltration and corresponding antitumor functions [242,243]. Candidate epigenetic biomarkers are expected to serve as informational biomarkers for patient stratification, thereby maximizing the chance of treatment success while minimizing side effects [244]. It is worth noting that epigenetic mechanisms are often associated with resistance to chemotherapy, radiation therapy, and hormone therapy [[245], [246], [247]]. Therefore, the application of epigenetic modifiers may resensitize patients with drug-resistant cancer to traditional therapies. However, further research is needed to better understand the mechanisms by which epigenetic drugs may develop resistance to peripheral therapies for cancer [[248], [249], [250]].

There is compelling evidence that epigenetic regulation affects the interaction between cancer cells, immune cells, stromal cells, and the immune tumor microenvironment [251]. Therefore, epigenetic regulation itself can serve as an intervention method to elicit moderate or even strong antitumor immunity. Lint et al. previously outlined the characteristics of successful anticancer immunotherapy, including the concomitant enhancement of innate and adaptive immunity [[252], [253], [254], [255]]. Therefore, a reasonable strategy for further enhancing the effectiveness of immunotherapy is to combine certain epigenetic modulators with one or more classical immunotherapy regimens, such as cancer vaccines, immune-checkpoint-inhibitor (ICI), oncolytic viruses, chimeric antigen receptor (CAR)-T cells, T cells expressing an engineered T cell receptor (TCR-T cells) cells, or other novel immunostimulators. Most human solid cancers are immunologically cold tumors, which are difficult to treat with immunotherapy [256]. In this case, epigenetic drugs can induce ICD and transform cold tumors, and may even be combined with other immunotherapies to overcome certain adverse reactions and prevent acquired resistance to single dose immunotherapy [257,258]. Currently, some combination drugs have shown early prospects in clinical research [[259], [260], [261]]. In the future, identifying the most important epigenetic targets in cancer and immune cells will be crucial to improve antitumor immunity and develop optimal combination strategies for treating patients with advanced solid cancer [43,262,263].

The ultimate aim of epigenetic therapy is to alleviate or treat diseases by altering gene expression. Despite its wide use [[264], [265], [266]], reversible hematological dose-limiting toxicity is often observed in patients undergoing epigenetic therapy. Wider application of functional genomics screening to systematically interrogate epigenetic dependencies in immune cells may help predict phenotypes that may be harmful to antitumor immunity [267]. The importance of genes encoding epigenetic regulatory factors for the survival of cancer cells has been identified through the unbiased genome-wide loss-of-function screening. In contrast, previous studies on the role of epigenetic regulators in regulating the development and function of immune cells have typically used small molecules to suppress individual genes or to generate time-consuming and expensive transgenic mouse models. Genome-level genetic screening in primary immune cells is hindered due to the inability to cultivate sufficient identified immune subsets in vitro and/or the technical challenges of gene editing and large-scale cell therapy in primary cells, such as CAR-T cells [268,269]. Of course, due to the development of genomics and sequencing technology, a variety of single cell or small number of cell research techniques have been applied to the tumor immune environment, which is of great significance for clinical research and new drug development. More importantly, based on the discovery of genomics, applying the powerful clustered regularly interspaced short palindromic repeats-CRISPR-associated 9 (CRISPR-Cas9) gene editing technology to the primary immune cell population is expected to accelerate these studies [270].

In summary, the combination of epigenetic therapy and immunotherapy is rapidly becoming a new paradigm for treating cancer [271]. Looking ahead, the improvement in the specificity and affinity of current epigenetic therapies, as well as the development of small molecules targeting a broader range of epigenetic and immune targets, coupled with new next-generation sequencing and immune technologies may provide more insights and opportunities for rational combinations [272]. Furthermore, the analysis of the transcriptome of tumor-infiltrating immune cells at the single cell level has greatly enhanced our understanding of the mechanisms of immune checkpoint inhibitors [[273], [274], [275]]. Similarly, we anticipate that multiple and temporal analysis of the epigenome of immune cells in the tumor microenvironment at single cell resolution will further refine the basic epigenetic processes supporting antitumor immunity. In summary, the ability to harness these complex biological interactions will provide exciting opportunities for new and improved cancer treatment interventions [276,277].

At present, there are more than 50 epigenetic targets under drug development, but the number of marketed drugs is limited and the types of tumors they can adapt to are also very limited, mainly used in hematological tumors. Due to the use of the strategy of targeting a single enzyme single site in the development of kinase drugs, there are still limitations in drug development. This article discussed the target innovation and strategic changes of various types of epigenetic functional elements, and look forward to new drug development directions.

## CRediT author statement

**Nian-nian Li, Deng-xing Lun, Ningning Gong** and **Gang Meng**:Methodology, Formal analysis, Investigation, Data curation; **Xin-ying Du, He Wang, Xiangxiang Bao, Xin-yang Li, Ji-wu Song, Kewei Hu, Lala Li, Si-ying Li, Wenbo Liu, Wanping Zhu, Yunlong Zhang, Jikai Li, Ting Yao, Leming-Mou, Xiaoqing Han** and **Furong Hao**: Conceptualization, Investigation, Data curation, Writing - Reviewing and Editing; **Yongcheng Hu, Lin Liu, Hongguang Zhu, Yuyun Wu** and **Bin Liu:** Resources, Writing - Reviewing and Editing, Data Curation, Visualization, Investigation, Formal Analysis, Validation, Software, Supervision, Funding acquisition.

## Declaration of competing interest

The authors declare that there are no conflicts of interest.
